# Nutritional and lipidomics biomarkers of docosahexaenoic acid-based multivitamin therapy in pediatric NASH

**DOI:** 10.1038/s41598-018-37209-y

**Published:** 2019-02-14

**Authors:** Pierangelo Torquato, Danilo Giusepponi, Anna Alisi, Roberta Galarini, Desirée Bartolini, Marta Piroddi, Laura Goracci, Alessandra Di Veroli, Gabriele Cruciani, Annalisa Crudele, Valerio Nobili, Francesco Galli

**Affiliations:** 1 Department of Pharmaceutical Sciences, University of Perugia, Perugia, Italy; 2 Istituto Zooprofilattico Sperimentale dell’Umbria e delle Marche (IZSUM), Perugia, Italy; 3 Research Unit of Molecular Genetics of Complex Phenotypes, Bambino Gesù Children’s Hospital, Rome, Italy; 4 Department of Chemistry, Biology and Biotechnology, University of Perugia, Perugia, Italy; 5 Hepatometabolic Unit, Bambino Gesù Children’s Hospital, Rome, Italy; 6 Department of Pediatrics, Sapienza University of Rome, Rome, Italy

## Abstract

Two recent randomized controlled trials demonstrated improved radiographic, histological and hepatometabolic cues of non-alcoholic steatohepatitis (NASH) in pediatric patients treated with the ω-3 fatty acid docosahexaenoic acid (DHA) in combination with vitamin D (VD) or with choline (CHO) and vitamin E (VE), the DHA-VD and DHA-CHO-VE trials, respectively). In the present study we verified the nutritional compliance to these DHA-based multivitamin treatments; lipidomics biomarkers of the reported outcome on NASH indicators were also investigated. Samples were obtained from 30 biopsy-proven pediatric NASH patients of the DHA-CHO-VE trial randomized in multivitamin treatment group and placebo group (n = 15 each), and from 12 patients of the treatment group of the DHA-VD trial. All patients underwent 6-month therapy plus 6 months of follow-up. Plasma samples and clinical data were obtained at baseline and at the end of the study (12 months). Selected biomarkers included the free form of DHA and other ω-3 fatty acid arachidonic acid (AA), indices of the vitamin E status, and some hepatic metabolites of these lipids. Radiographic and histological improvements of treated patients were associated with increased concentrations of DHA, α-linolenic acid and α-tocopherol (i.e. VE), and with decreased AA that was also investigated in complex lipids by untargetd lipidomics. As a result a significantly lowered AA/DHA ratio was observed to represent the main indicator of the response to the DHA-based therapy. Furthermore, baseline levels of AA/DHA showed strong association with NAS and US improvement. A stable correction of DHA AA metabolism interaction is associated with the curative effect of this therapy and may represent a key nutritional endpoint in the clinical management of pediatric NASH.

## Introduction

Non-alcoholic fatty liver disease (NAFLD) is now representing one of the main chronic liver diseases of both adults and children worldwide^1,2^. Its prevalence in the global pediatric population has been estimated to range between 3 and 12%, with a rise up to 90% in obese children^2^.

NAFLD encompasses a spectrum of liver conditions, ranging from simple steatosis, a relatively benign and reversible condition, to a progressive and persistent activation of inflammatory and fibrotic processes of non-alcoholic steatohepatitis (NASH)^3^. Although the “juvenile form” of the disease is often less severe^2^, it remains a major risk factor for insulin resistance and its metabolic comorbidity^4^, as well as for cirrhosis and hepatocellular carcinomas^5,6^, with epidemiology projections that identify this as the most common cause of liver transplantation in children in the near future^7^.

NAFLD is fundamentally a severe lipid disorder of the liver with consequences on storage mechanisms and metabolism of lipoproteins, free fatty acids (FFA) and fat-soluble micronutrient vitamins^8–10^. The intra-hepatic accumulation of lipids and their abnormal metabolism cause a series of lesions identified with the term of lipotoxicity that are believed to play a major role in generating the irreversible liver damages of NASH [recently reviewed in^11^].

In such an alarming scenario, a number of attempts have been made to diagnose and cure the metabolic and inflammatory cues of NAFLD and NASH. These include interventions with micronutrients and vitamins^12^. Docosahexaenoic acid (DHA), choline (CHO) and the fat-soluble vitamin E (VE) and D (VD), have been among the most investigated in recent times. Promising findings have been obtained with DHA, an ω-3 FA with effects on the immune modulation and inflammatory homeostasis of tissues, which has been increasingly investigated as a therapeutic agent in NAFLD and NASH [reviewed in^10,13^]. Recent studies^14–16^ and two consecutive clinical trials^17,18^ carried out in pediatric NAFLD and NASH demonstrated the efficacy of DHA to improve laboratory or histology hallmarks of liver damage when administered either alone or in combination with other micronutrients, such as the parent ω-3 compound eicosapentaenoic acid (EPA), fat soluble vitamins and CHO. In the two randomized clinical trials carried out in pediatric NASH patients, DHA therapy was investigated with the same study design and end-points, administrating a dose of 250 or 500 mg/day of this micronutrient combined with VD^17^ or CHO + VE^18^, respectively. An improved NAFLD activity score (NAS) was obtained as an effect of this therapy in both the two studies. Other positive findings included improvements of liver echogenicity, some laboratory indices of metabolic syndrome and specific liver biopsy hallmarks, such as steatosis and ballooning in the DHA-CHO-VE study^18^, and steatosis, hepatic stellate cell activation and fibrillar collagen levels in the DHA-VD study^17^.

Our aim in the present study is to extend the investigation of these DHA-based multivitamin RCTs^17,18^ to nutritional and lipid metabolism biomarkers that may help to explain mechanistic aspects of the reported radiographic and histological outcomes; these include targeted indicators of the patient’ vitamin status and compliance to the treatment, as well as biomarkers of the hepatic metabolism of the administered micronutrients and of the ω-6 arachidonic acid (AA), the latter is expected to represent an elective target of DHA-based interventions in these patients^10^. Most of the nutritional and lipidomics (i.e. nutrigenomics) biomarkers proposed in the present study have been investigated for the first time in obese NASH pediatric patients.

## Results

### Clinical data

Clinical and routine laboratory data of pediatric patients included in this post-hoc laboratory investigation of DHA-VD and DHA-CHO-VE trials^17,18^ are reported in Supplementary Table S1A–D; patient groups were: (i) DHA-CHO-VE group (n = 15) and its corresponding placebo group (n = 15); (ii) DHA-VD group (n = 12); (iii) age-matched healthy controls (n = 10). The primary endpoint US significantly improved in DHA-based treatment subgroups and also in placebo group (data of DHA-based treatment subgroups merged together are reported in Supplementary Figure S1). The endpoint NAS was improved after treatment in both the subgroups of patients selected in the two RCTs^17,18^, but not in the placebo group. Supplementary Table S1D shows corresponding data from a group of age-matched healthy controls. LDL and fasting blood glucose and (p = 0.05) decreased in the DHA-CHO-VE subgroup, but not in DHA-VD subgroup, by the effect of the treatment (Supplementary Table S1A).

Comparing DHA-CHO-VE and DHA-VD subgroups no significant differences were observed in the endpoint NAS at either baseline (T0) and end-of-the-study (T1) evaluation (Supplementary Table S1B), suggesting similar efficacy of the two DHA-based therapy protocols. Significant differences between the two subgroups were: subject age (2 years higher in DHA-CHO-VE subgroup), and US and LDL cholesterol that were lower in DHA-CHO-VE subgroup at both T0 and T1, and triglycerides that were lowered at T1 evaluation (Supplementary Figure S1C). None of these parameters (i.e. age, height, WC, BMI, and cholesterol and triglyceride fractions) were found to represent confounding variables of correlations assessed between lipidomics parameters and endpoints investigated in this study with different regression models (described in the next sections).

Compared to healthy controls, NASH patients of the different study cumulatively assessed at baseline, showed increased height, body weight, BMI, ALT, and blood glucose, and decreased LDL and HDL cholesterol (Supplementary Table S1). Of these differences between healthy controls and NASH patients, HDL and blood glucose were corrected at the end of the study.

Cumulating data of the two DHA-based treatments (DHA-CHO-VE + DHA-VD) and comparing them with data of placebo group (Table 1), resulted in the identification at baseline evaluation (T0) of lowered levels of height and uric acid, and increased US levels, Vitamin D concentrations significantly increased post-treatment in the DHA-VD subgroup of patients (Table 1). Choline levels assessed in the subgroup of patients of the DHA-CHO-VE trial significantly increased in the placebo, but not in the treatment group (Table 1).

**Table 1:** Clinical and laboratory parameters in the DHA-based multivitamin treatment and placebo group.

*Anthropometry and general characteristics*	DHA-based multivitamin group (n = 27)									
	Baseline evaluation (T0)				Follow-up (T1)					
	Mean ± SD	Median	Interval (Min-Max)	P (vs. Placebo arm T0)	Mean ± SD		Median	Interval (Min-Max)		P (vs. DHA arm T0)
*Sex*	15/12									
*Age* (*yrs*)	12.5 ± 2.4	12.5	9.0–17.0	0.06	13.5 ± 2.4		13.5	10.0–18.0		0.07
*Height* (*m*)	1.5 ± 0.1	1.5	1.3–1.7	**0.01**	1.5 ± 0.1		1.5	1.3–1.8		0.25
*WC* (*cm*)	85.7 ± 7.3	86.5	70.0–101.0	0.06	89.0 ± 9.0		90.5	72.0–106.5		0.08
*Weight* (*kg*)	64.3 ± 15.4	65.5	33.5–84.6	0.35	67.4 ± 14.9		67.7	37.3–94.5		0.23
*BMI*	27.4 ± 3.6	29.0	19.8–32.8	0.25	27.2 ± 4.4		28.6	17.2–37.3		0.43
***Laboratory parameters***										
*Tot*. *Ch* (*mg*/*dL*)	158.5 ± 29.7	152.5	116.0–257.0	0.46	155.6 ± 24.4		152.5	103.0–219.0		0.35
*HDL* (*mg*/*dL*)	46.5 ± 7.6	47.5	34.0–65.0	0.40	46.1 ± 10.5		46.5	28.0–81.0		0.44
*LDL* (*mg*/*dL*)	99.8 ± 27.0	95.0	30.0–156.0	0.36	93.2 ± 31.6		93.5	35.0–151.0		0.21
*Triglycerides* (*mg*/*dL*)	110.1 ± 50.7	110.5	35.0–224.0	0.10	111.7 ± 63.0		93.5	29.0–270.0		0.46
*Uric Acid* (*mg*/*dL*)	5.5 ± 1.2	5.5	3.1–7.4	**0.03**	5.6 ± 1.5		5.8	3.3–9.8		0.37
*AST* (*UI*/*L*)	30.8 ± 12.4	28.0	12.0–65.0	0.27	30.8 ± 18.1		25.0	17.0–85.0		0.50
*ALT* (*UI*/*L*)	40.3 ± 26.2	35.0	17.0–135.0	0.14	32.8 ± 23.1		22.0	16.0–110.0		0.14
*GGT* (*UI*/*L*)	17.9 ± 11.7	14.5	6.0–65.0	0.32	21.1 ± 15.3		17.0	6.0–79.0		0.20
*Blood glucose* (*mg*/*dL*)	84.8 ± 7.4	84.0	71.0–102.0	0.25	83.7 ± 10.5		84.0	67.0–122.0		0.33
*Insulin* (*mU*/*L*)	22.5 ± 21.1	16.5	0.4–104.4	0.45	17.8 ± 11.5		16.0	6.3–65.4		0.16
*HOMA index*	4.7 ± 4.3	3.7	0.1–20.6	0.47	3.6 ± 2.1		3.3	1.2–12.3		0.12
*Choline* (*μM*)*	9.6 ± 6.6	6.2	3.1–23.6	0.09	8.9 ± 4.9		7.3	3.8–19.8		0.74
*Vitamin D* (*ng*/*ml*)*^*	14.3 ± 5.6	16.5	1.8–20.0	0.13	22.56.1		21.5	14.7–34.5		**0.00**
***US and NAS***										
*US*	2.8 ± 0.9	3.0	1.04.0	**0.00**	1.4	0.9	1.0	0.0	3.0	**0.00**
*NAS*	4.5 ± 1.0	5.0	2.0–6.0	0.49	2.7	1.5	3.0	0.0	5.0	**0.00**
***Anthropometry and general characteristics***	**Placebo group (n** = **15)**									
	**Baseline evaluation (T0)**				**Follow-up (T1)**					
	**Mean** ± **SD**	**Median**	**Interval (Min-Max)**		**Mean** ± **SD**		**Median**	**Interval (Min–Max)**		**P (vs. Placebo arm T0)**
*Sex*	8/7									
*Age* (*yrs*)	13.7 ± 2.1	13.0	10.0–17.0		14.7 ± 2.1		14.0	11.0–18.0		0.10
*Height* (*m*)	1.6 ± 0.1	1.6	1.5–1.7		1.6 ± 0.1		1.6	1.5–1.7		0.50
*WC* (*cm*)	90.1 ± 10.1	92.0	69.0–109.0		89.1 ± 9.9		90.0	69.0–109.0		0.39
*Weight* (*kg*)	66.4 ± 15.9	68.5	35.3–85.5		66.5 ± 16.1		68.5	35.3–85.0		0.49
*BMI*	28.4 ± 5.6	28.9	20.3–43.7		28.2 ± 5.5		28.9	20.3–43.7		0.45
***Laboratory parameters***										
*Tot*. *Cholesterol* (*mg*/*dL*)	157.5 ± 31.8	151.0	125.0–257.0		146.7 ± 16.9		142.0	125.0–177.0		0.13
*HDL* (*mg*/*dL*)	45.9 ± 7.0	47.0	36.0–62.0		48.4 ± 7.1		48.0	39.0–69.0		0.17
*LDL* (*mg*/*dL*)	103.5 ± 39.5	94.0	51.0–196.0		92.9 ± 31.2		93.0	53.0–189.0		0.21
*Triglycerides* (*mg*/*dL*)	88.9 ± 47.1	80.0	35.0–192.0		81.4 ± 35.7		69.0	44.0–170.0		0.31
*Uric Acid* (*mg*/*dL*)	6.9 ± 3.5	5.6	3.6–15.0		6.9 ± 3.5		5.6	3.6–15.0		0.50
*AST* (*UI*/*L*)	34.0 ± 21.5	25.0	14.0–102.0		37.3 ± 42.7		24.0	18.0–189.0		0.39
*ALT* (*UI*/*L*)	55.6 ± 60.2	25.0	17.0–246.0		32.9 ± 18.0		29.0	16.0–79.0		0.09
*GGT* (*UI*/*L*)	19.8 ± 14.0	17.0	10.0–68.0		20.5 ± 10.1		18.0	12.0–51.0		0.44
*Blood glucose* (*mg*/*dL*)	83.1 ± 7.9	80.0	73.0–99.0		81.0 ± 7.1		79.0	71.0–94.0		0.23
*Insulin* (*mU*/*L*)	23.3 ± 16.8	17.7	7.3–60.4		21.6 ± 13.5		15.5	11.5–59.8		0.38
*HOMA index*	4.8 ± 3.6	3.6	1.6–13.6		4.3 ± 2.8		3.5	2.2–12.8		0.34
*Choline* (*μM*)*	6.4 ± 3.8	4.9	2.6-0.4		7.5 ± 4.1		6.3	3.0–20.4		**0.04**
*Vitamin D* (*ng*/*ml*)*^*	13.9 ± 2.9	13.9	9.1–18.2		14.2 ± 2.6		13.9	11.0–18.1		0.42
***US and NAS***										
*US*	2.0 ± 0.8	2.0	1.0–3.0		1.5 ± 0.6		1.0	1.0–2.5		**0.02**
*NAS*	4.5 ± 1.4	5.0	2.0–7.0		4.2 ± 1.1		4.0	2.0–6.0		0.28

*Investigated in the DHA-CHO-VE group.

^Investigated in the DHA-VD group; Significant differences were as P values in bold (≤0.05).

*Investigated in the DHA-CHO-VE group.

^Investigated in the DHA-VD group; Significant differences were as P values in bold (≤0.05).

### Targeted lipidomics of ω-3 species and the ω-6 fatty acid AA

**Figure 1:**
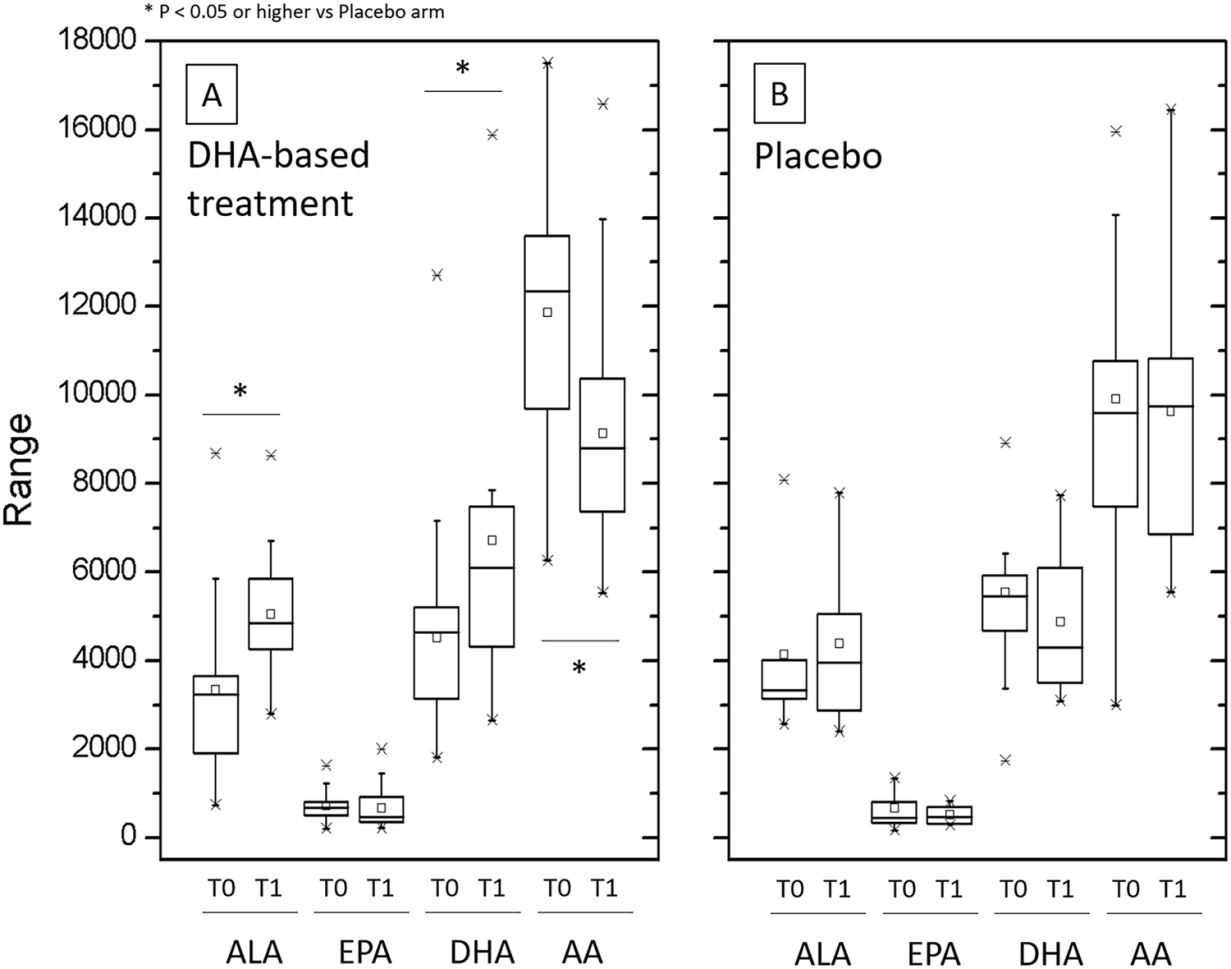
Plasma concentrations of the polyunsaturated fatty acids ALA, EPA, DHA and AA in pediatric NASH patients treated with DHA-based multivitamin therapy or placebo. The free form of these polyunsaturated fatty acids was measured at baseline (T0) and at the end of follow-up (T1) in the groups of patients treated with the DHA-based multivitamin formulations (DHA treatment subgroups are merged) or with placebo (panel A and B, respectively) as described in the Boxe charts show minimum and maximum values (asterisks), mean and median values of the distribution interval (square symbol and horizontal line within the box, respectively), 25–75 percentile (upper and lower margin of the boxes) and 5–95 percentile (whisker margins) of the interquartile range.

The multivitamin intervention significantly increased DHA concentrations in both the treatment subgroups (data are shown separate in Supplementary Table S1 and combined in Table 2 and Fig. 1A); similarly, ALA, but not EPA, concentrations increased in these subgroups by the effect of the treatment, while AA decreased. Conversely, mean concentrations of DHA slightly decreased, and ALA and AA remained almost unchanged in the placebo group. A positive relationship between DHA and AA, and DHA and ALA concentrations was observed in all experimental groups at baseline (Supplementary Figure S2). Such relationship was also maintained at the end of the study, but regression line slope significantly changed in the different groups by the effect of the DHA-based multivitamin therapy (Supplementary Figure S2).

Data analysis carried out by LRA confirmed the significant effect of the DHA-based multivitamin treatment on the categorical variables US and NAS, and highlighted the positive relationship existing between these variables and DHA levels in the DHA-based treatment group (Supplementary Figure S3). Strongest associations were observed in the case of US variable. Much weaker and not significant was the relationship of US and NAS with AA. Conversely, in the placebo group US and NAS variables showed a significant negative correlation with DHA at baseline evaluation that underwent to minor, but significant, modification at the end of the study (Supplementary Figure S3).

When a multivariable (multiple regression) model was used to assess the correlation of these targeted lipidomics indicators (set as independent variables in the model) with other biomarkers and parameters investigated in this study (Supplementary Table S2), DHA and its derived variable AA/DHA (discussed later) showed strong significant relationships with ω-3 fatty acids, AA, AA/EPA, and especially with α-TOH and some of its metabolites, such as α-CEHC and α-13′-OH that are presented in detail later.

In the DHA-VD group, nor DHA neither other PUFAs correlated with VD levels. When the latter were set as independent variables, significant correlations were observed only with BMI and triglycerides.

All these correlations were not affected by possible confounding variables, such as age, BMI and WC (not shown).

### Enzymatic metabolites of AA

Absolute concentrations of the vasoactive eicosanoid 20-HETE significantly decreased along with the levels of its AA precursor by the effect of the DHA-based multivitamin treatment (Table 2 and Suppl. Figure S2), and especially in the DHA-CHO-VE subgroup, reaching levels even lower than in healthy control subjects (Supplementary Table S1). The ω-oxidation product 20-COOH-AA slightly increased after DHA-based treatment. On the contrary, the absolute and AA-corrected concentrations of these metabolites slightly decreased in the placebo group during the study (Table 2).

**Table 2:** Polyunsaturated free fatty acids (ω-3 series and arachidonic acid) in the DHA-based multivitamin treatment and placebo group.

*Parameters*	DHA-based multivitamin group (n = 27)							
	Baseline evaluation (T0)				Follow-up (T1)			
	Mean ± SD	Median	Interval (Min-Max)	P (vs. Placebo arm T0)	Mean ± SD	Median	Interval (Min-Max)	P (vs. DHA arm T0)
***ω***-**3** ***fatty acids*** **(*nM*)**								
*ALA*	3338.0 ± 1759.7	3245.9	731.7–8678.2	0.11	5049.9 ± 1268.0	4876.2	2793.3–8628.2	**0.00**
*EPA*	713.6 ± 353.0	668.0	212.4–1623.5	0.34	663.4 ± 430.1	496.4	221.2–2005.8	0.33
*DHA*	4524.4 ± 2176.0	4631.3	1808.1–12706.9	0.09	6727.8 ± 3527.8	6167.5	2651.3–15879.3	**0.01**
***Arachidonic acid and metabolites*** **(** ***nM*** **)**								
*AA*	11865.0 ± 3301.7	12338.9	6267.8–17507.5	**0.05**	9124.6 ± 2599.6	8841.9	5534.1–16580.1	**0.00**
*20*-*COOH*-*AA*	9.9 ± 6.4	9.0	1.4–30.5	**0.03**	10.7 ± 6.7	10.5	2.1–30.8	0.34
*20*-*COOH*-*AA*/*AA* (*x 1000*)	0.9 ± 0.8	0.7	0.1–4.0	**0.02**	1.2 ± 0.8	1.0	0.2–3.1	0.10
*20*-*HETE*	18.1 ± 6.9	18.3	7.5–31.7	0.49	12.3 ± 3.8	11.8	5.1–23.7	**0.00**
*20*-*HETE*/*AA* (*x 1000*)	1.7 ± 0.9	1.5	0.7–4.0	0.15	1.4 ± 0.6	1.3	0.6–2.8	0.14
***AA*** **/** ***EPA***	19.8 ± 9.0	18.8	6.0–44.6	0.25	17.7 ± 8.5	17.2	6.3–39.8	0.20
***AA*** **/** ***DHA***	2.9 ± 1.0	2.6	1.3–5.0	**0.00**	1.6 ± 0.6	1.5	0.7–2.8	**0.00**
***Parameters***	**Placebo group (n** = **15)**							
	**Baseline evaluation (T0)**				**Follow-up (T1)**			
	**Mean** ± **SD**	**Median**	**Interval (Min-Max)**		**Mean** ± **SD**	**Median**	**Interval (Min-Max)**	**P (vs. Placebo arm T0)**
***ω***-***3 fatty acids*** **(*nM*)**								
*ALA*	4130.3 ± 1822.9	3426.0	2563.0–8080.5		4380.3 ± 1764.6	3975.2	2404.0–7785.3	0.37
*EPA*	659.5 ± 391.2	529.9	169.7–1343.7		519.2 ± 200.2	510.0	286.8–824.9	0.14
*DHA*	5537.0 ± 1890.5	5490.0	1743.9–8920.3		4885.4 ± 1556.5	4358.3	3089.1–7728.4	0.18
***Arachidonic acid and metabolites*** **(** ***nM*** **)**								
*AA*	9911.9 ± 3523.5	9593.8	2997.4–15959.3		9632.5 ± 3214.6	9848.6	5543.5–16451.5	0.42
*20*-*COOH*-*AA*	13.9 ± 4.9	13.3	7.1–21.3		10.8 ± 6.4	8.6	3.6–24.8	0.10
*20*-*COOH*-*AA*/*AA* (*x 1000*)	1.6 ± 1.2	1.3	0.7–5.1		1.2 ± 0.7	1.0	0.3–2.6	0.12
*20*-*HETE*	18.0 ± 8.2	17.3	6.6–31.8		13.7 ± 6.1	12.5	6.6–24.7	0.08
*20*-*HETE*/*AA* (*x 1000*)	2.0 ± 1.1	2.0	0.7–4.1		1.5 ± 0.6	1.3	0.6–3.0	0.07
*AA*/*EPA*	17.9 ± 6.6	17.9	9.5–28.4		19.7 ± 5.3	20.4	9.9–29.6	0.23
*AA*/*DHA*	1.8 ± 0.3	1.8	1.3–2.3		2.0 ± 0.3	2.0	1.6–2.7	0.07

Significant differences were as P values in bold (≤0.05).

### The AA/DHA ratio

**Figure 2:**
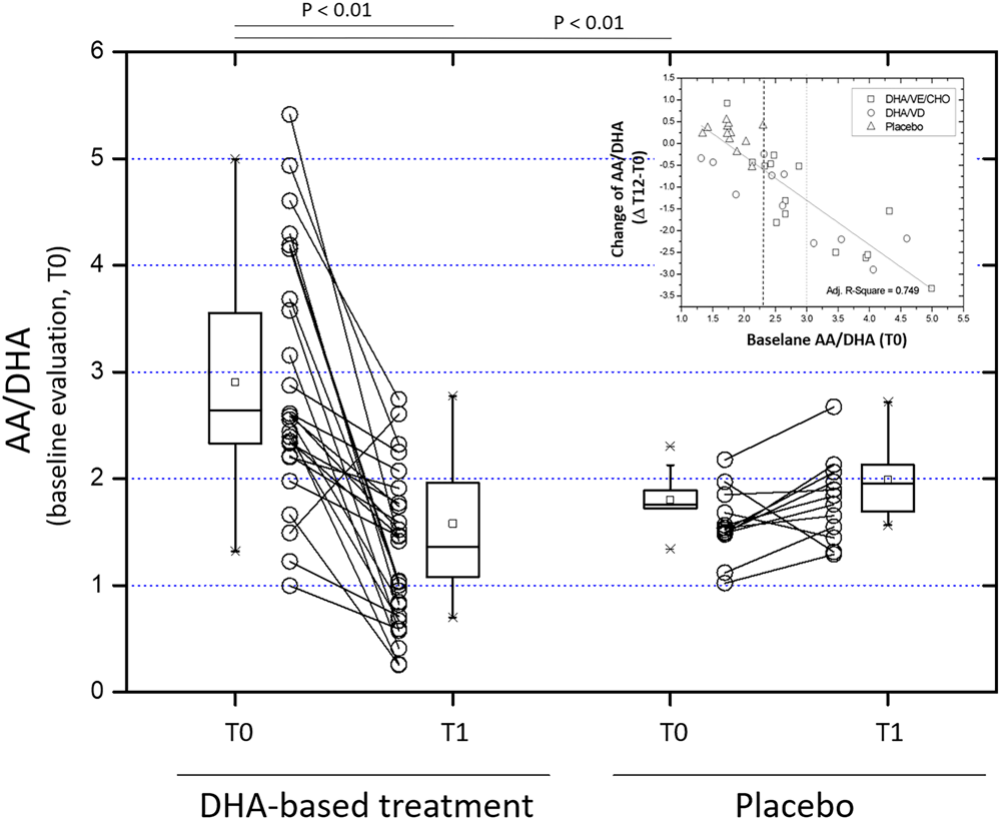
AA/DHA ratio in pediatric NASH patients treated with DHA-based multivitamin therapy (left) or placebo (right). The ratio between the free form of the polyunsaturated fatty acids AA and DHA is shown at baseline (T0) and at the end of follow-up (T1), and as treatment-induced changes (Δ T12-T0) and their relationship with baseline (T0) values of AA/DHA ratio were assessed scatter plot and linear regression analysis data are shown in the insert on the top-right corner). In this chart, the dashed line at the intersection with the abscissa shows the upper limit of AA/DHA interval of data of placebo group.

As a consequence of the changes on PUFA species described above in the previous sections, AA/DHA markedly decreased in DHA-treated patients of the two trials (Table 2 and Fig. 2); when individual data were considered, AA/DHA was found to decrease in 26 out of 27 patients of the DHA-based treatment group (96%) and only in 2 of the 15 patients in the placebo group (13%) (Fig. 2). Baseline AA/DHA ratio was significantly higher in the treatment group compared with the placebo group (Table 2 and Fig. 2).

**Figure 3:**
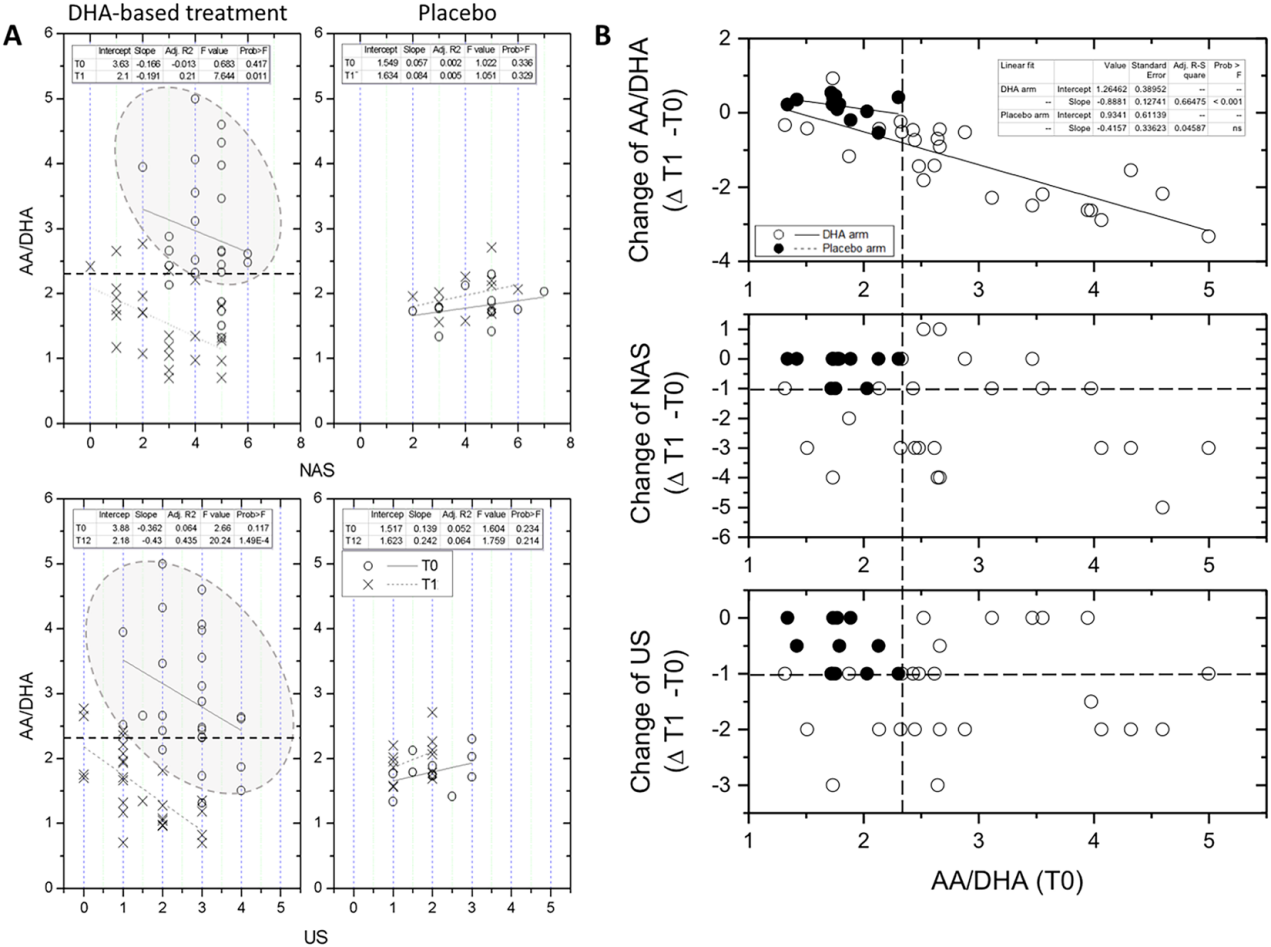
Regression analysis between the AA/DHA ratio and radiographic and histological endpoints (US or NAS, respectively) and the AA/DHA ratio in pediatric NASH patients treated with DHA-based multivitamin therapy (left) or placebo (right). (**A**) Logistic regression analysis was used to assess the relationship between the AA/DHA ratio and the categorical variables NAS (upper panels) and US (lower panels) in the two time-points of the study (**B**) The regression model was then used to assess the relationship between baseline (T0) AA/DHA and the change of these radiographic and histological endpoints during the study by the effect of the proposed treatment: upper panel = (Δ T1-T0 of AA/DHA; see also insert to Fig. 2), middle panel = (Δ T1-T0 of US), and lower panel (Δ T1-T0 of NAS). Dashed lines at the intersection with the orthogonal axes identify the upper limits in the interval of data in the placebo group. The DHA-based treatment group included DHA-VD and DHA-CHO-VE subgroups merged together.

Highly significant correlations were observed between AA/DHA (the independent variable) and the radiographic and histological endpoints NAS and US (p ≤ 0.01; Fig. 3A). Such relationship assessed by LRA was even more significant than in the case of DHA or AA (Supplementary Figure S3). Again, in the case of US parameter the regression model provided a complete separation of pre-treatment and post-treatment data points - highlighted by the 95 percentile ellipsoid of the lower panel of Fig. 3A. Less efficient was the separation effect in the case of NAS, with an overlap between pre- and post-treatment data in 6 patients.

These findings suggested us to extend the application of this regression model to calculate the power of AA/DHA indicator of response for US and NAS parameters. Baseline levels of AA/DHA showed a very significant relationship with its change that the treatment produced at the end of the study (insert to Fig. 2 and upper panel of Fig. 3B). A negative relationship of baseline AA/DHA with the categorical variables NAS and US was also observed (Fig. 3, middle and lower panels). Using such regression data and arbitrarily selecting the first levels of response for NAS and US endpoints as clinically relevant (reduction ≥−1), 14 out of 27 treated patients (52%) were identified to gather out of the area of distribution of placebo group data. Such discrimination between placebo and DHA-treated patients occurred at a baseline AA/DHA value ≥2.35. This threshold value identifies responders of the DHA-based multivitamin therapy in this study excluding false positives and negatives (Fig. 3B; further discussed later).These findings suggest that the AA/DHA ratio could be firther investigated for an application as nutritional biomarker in screening protocols to identify candidate patients that that may benefit of DHA therapy. Other applications obviously include verification of the nutritional compliance and monitoring of patients during the intervention.

### Vitamin E status and metabolism

Absolute and cholesterol-corrected concentrations of α-TOH increased in DHA-treated patients, but not in placebo group (Table 3). At the same time, γ-TOH decreased by the effect of the treatment. These effects on the two main forms of vitamin E^9^ were also observed when the two subgroups of patients of the DHA-VD and DHA-CHO-VE trials were assessed separate (Supplementary Table S1) thus suggesting an effect of DHA-based therapy on this vitamin independent of VE co-supplementation.

**Table 3:** Vitamin E parameters in the groups of patients treated with DHA-based multivitamin therapy or placebo.

*Parameters*	DHA-based multivitamin group (n = 27)							
	Baseline evaluation (T0)				Follow-up (T1)			
	Mean ± SD	Median	Interval (Min-Max)	P (vs. Placebo arm T0)	Mean ± SD	Median	Interval (Min–Max)	P (vs. DHA arm T0)
*α*-*TOH*(*nM*)	18010.2 ± 7873.8	15948.1	6765.7–36922.8	0.37	22244.5 ± 8773.4	22303.3	9795.2–39020.6	**0.04**
*α*-*TOH*/*Tot lipids* (*nmol*/*mg*)	7.08 ± 2.91	6.98	2.50–13.57	0.34	8.50 ± 3.29	7.93	4.71–16.55	0.06
*α*-*TOH*/*Cho* (*nmol*/*mg*)	11.49 ± 4.29	11.15	4.60–20.98	0.25	14.50 ± 5.84	13.87	6.59–26.91	**0.02**
*α*-*TOH*/*TG* (*nmol*/*mg*)	21.76 ± 13.74	18.54	4.48–51.76	0.34	24.61 ± 13.09	22.55	8.06–56.33	0.23
*α*-*13'*-*OH* (*nM*)	14.1 ± 13.5	10.0	1.7–54.6	0.31	12.1 ± 12.6	7.8	3.7–64.9	0.30
*α*-*13'*-*OH* (*M3 isomer; nM*)	5.1 ± 5.6	3.7	0.2–26.9	0.22	3.6 ± 5.4	1.8	0.3–24.3	0.15
*α*-*13′*-*OH* (*M3 nM*)	19.1 ± 15.7	13.8	4.0–63.5	0.26	15.7 ± 16.7	9.8	5.2–82.7	0.23
*α*-*13'*-*COOH* (*nM*)	1.6 ± 0.7	1.4	0.5–2.7	0.38	1.6 ± 1.2	1.3	0.6–5.7	0.41
*α*-*CEHC* (*nM*)	15.6 ± 14.3	10.9	6.6–66.9	0.41	35.7 ± 27.7	30.3	8.1–143.8	**0.00**
*γ*-*TOH* (*nM*)	584.7 ± 271.3	544.3	219.2–1218.0	0.13	455.2 ± 229.2	382.7	175.3–933.6	**0.04**
*γ*-*TOH*/*Tot lipids* (*nmol*/*mg*)	0.46 ± 0.26	0.48	0.12–0.99	0.38	0.38 ± 0.24	0.35	0.11–1.24	0.11
*γ*-*TOH*/*Cho* (*nmol*/*mg*)	0.38 ± 0.17	0.38	0.11–0.88	0.16	0.30 ± 0.18	0.24	0.11–0.84	0.07
*γ*-*CEHC* (*nM*)	147.9 ± 80.2	137.2	53.0–342.5	0.43	135.0 ± 74.8	108.2	41.3–335.6	0.28
***Parameters***	**Placebo group (n** **=** **15)**							
	**Baseline evaluation (T0)**				**Follow-up (T1)**			
	**Mean** **±** **SD**	**Median**	**Interval (Min-Max)**		**Mean** **±** **SD**	**Median**	**Interval (Min-Max)**	**P (vs. Placebo arm T0)**
*α*-*TOH*(*nM*)	17114.0 ± 3675.6	16901.8	11325.9–24933.5		17926.6 ± 6595.6	16661.9	8174.7–32171.3	0.37
*α*-*TOH*/*Tot lipids* (*nmol*/*mg*)	6.64 ± 2.74	7.70	0.0–9.94		8.01 ± 3.44	8.00	3.49–15.25	0.15
*α*-*TOH*/*Cho* (*nmol*/*mg*)	10.43 ± 4.18	11.51	0.0–14.79		12.17 ± 4.58	12.08	5.41–22.66	0.18
*α*-*TOH*/*TG* (*nmol*/*mg*)	19.87 ± 9.55	21.38	0.0–30.25		25.58 ± 14.02	25.12	6.94–48.01	0.14
*α*-*13'*-*OH* (*nM*)	11.8 ± 10.3	9.3	1.7–42.8		10.9 ± 6.6	10.0	1.7–24.6	0.40
*α*-*13'*-*OH*(*M*_*3*_ *isomer: nm*)	3.7 ± 3.8	2.5	0.2–13.8		5.8 ± 5.9	2.9	0.4–19.3	0.17
*α*-*13*′-*OH + M3* (*nM*)	15.6 ± 13.6	11.5	2.0–56.6		16.7 ± 10.4	13.9	3.8–37.7	0.41
*α*-*13'*-*COOH* (*nM*)	1.6 ± 0.9	1.8	0.2–2.8		2.6 ± 1.3	2.3	1.1–5.0	0.03
*α*-*CEHC* (*nM*)	14.4 ± 16.0	10.9	2.6–59.6		14.0 ± 8.5	10.5	5.5–35.2	0.47
*γ*-*TOH* (*nM*)	485.5 ± 184.9	533.0	144.6–743.3		447.5 ± 133.6	438.5	239.1–717.8	0.28
*γ*-*TOH*/*Tot lipids* (*nmol*/*mg*)	0.44 ± 0.24	0.49	0.04–0.74		0.46 ± 0.29	0.49	0.13–1.20	0.40
*γ*-*TOH*/*Cho* (*nmol*/*mg*)	0.32 ± 0.13	0.34	0.06–0.52		0.30 ± 0.09	0.30	0.14–0.47	0.38
*γ*-*CEHC* (*nM*)	142.2 ± 118.1	92.1	34.6–378.2		141.6 ± 94.3	112.6	54.5–333.6	0.49

Significant differences were as P values (≤0.05) and are highlighted in bold.

Concentrations of short-chain tocopherol metabolites (i.e. CEHCs) followed those of their vitamin precursors with a significant increase of α-CEHC in the treatment group (Table 3 and Supplementary Table S1). Conversely, the long-chain metabolite α-13′-OH and its isomer M3 showed a trend toward a decrease after treatment; the opposite was found in the placebo group that showed increased levels of α-13′-COOH at the end of the study (Table 3).

Multiple regression analysis of data in DHA-treated patients showed that α-TOH/Cho levels (set as independent variable) significantly correlate with vitamin E metabolites and PUFAs, and also with lipid parameters and uric acid (Supplementary Table S2).

### Untargeted lipidomics

**Figure 4:**
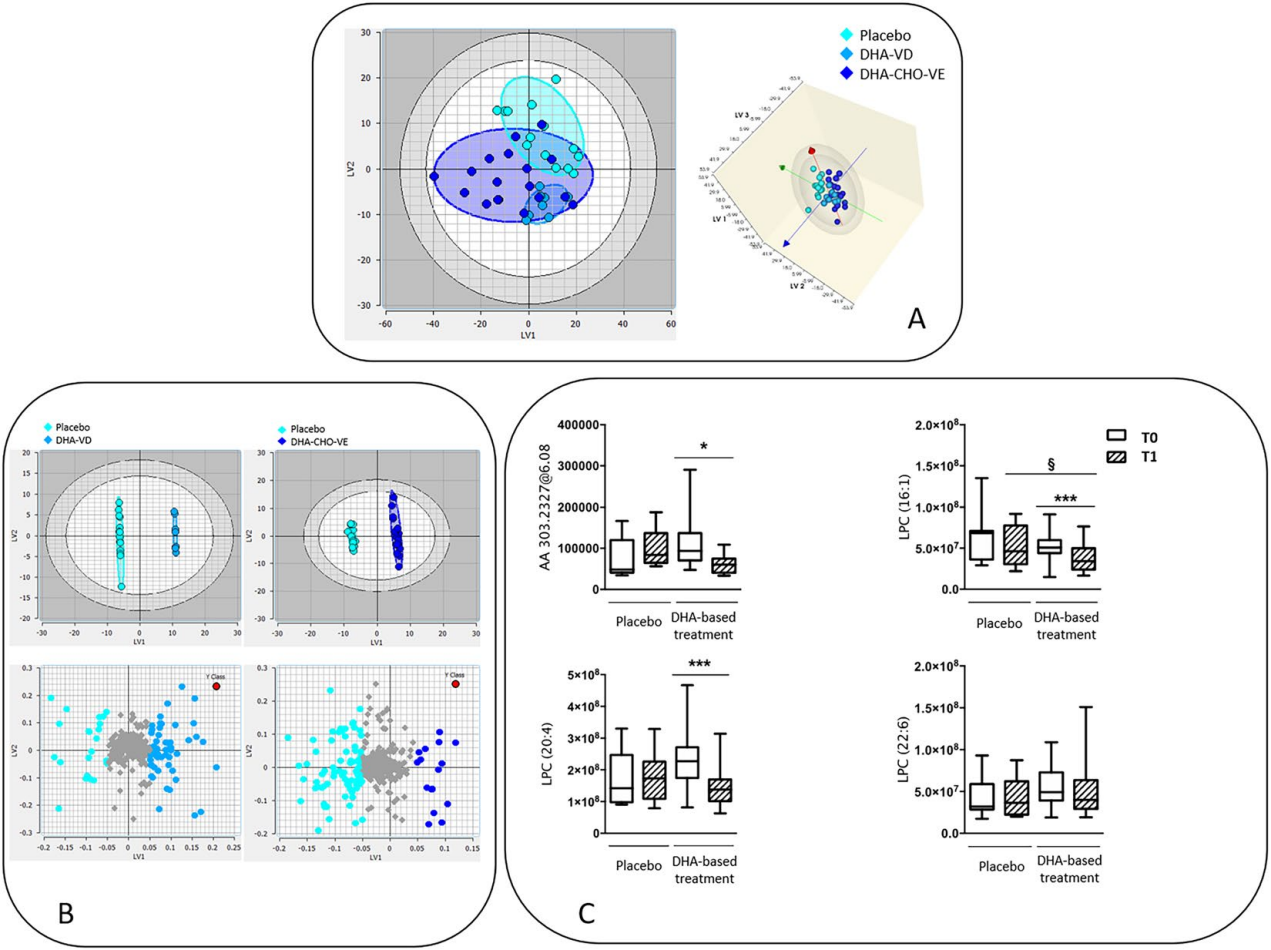
Untargeted lipidomics data in the subgroups of pediatric NASH patients treated with DHA-based multivitamin therapy or placebo. (**A**) Partial Least Squares Discriminant Analysis (PLS-DA) of lipidomics data in the subgroups: placebo, DHA-VD and DHA-CHO-VE. 2D and 3D score plot are shown (left and right panel, respectively). (**B**) Partial Least Squares Target Projection (PLS-TP) model carried out using the placebo group as a reference cluster for the comparison with DHA-VD subgroup (left upper score plot chart) and DHA-CHO-VE subgroup (right upper chart). Loading plots with the individual lipid species that differentiate the subgroups are shown in the lower panels. (**C**) Relative abundance of AA in the lipid species identified during the untargeted analysis (upper left chart) and of three LPC forms containing 16:1 (upper right chart) and 20:4 or 22:6 acyl residues (lower charts). Data were obtained by LC-MS/MS analysis and assessed by Lipostar software^52^ according with the procedure described in the text.

In an effort to verify if the changes observed on the FFA form of DHA and AA may correspond to changes of these PUFAs on complex lipids, an untargeted approach was adopted to assess the lipidome of patients treated with DHA-based multivitamin therapy or placebo. Multivariate analysis was used to evaluate significant differences between these groups (Fig. 4).

A first Partial Least Squares Discriminant Analysis (PLS-DA) was performed to evaluate whether or not the approach could distinguish the three groups (placebo, DHA-VD and DHA-CHO-VE) based on their lipid profile. The scores plot in Fig. 4A shows that placebo and DHA-VD group are different (no overlap between the two regions identified by the ellipsoids), while DHA-CHO-VE group is spread over a larger area, partially overlapping with the other clusters. To independently investigate the effect of the two treatments (i.e. DHA-VD vs. DHA-CHO-VE) on the patient’ lipidome, a Partial Least Squares Target Projection (PLS-TP) model was implemented using the placebo group as a reference cluster (Fig. 4B). The model provided a clear discrimination of the compared groups of patients in both the two DHA-based trials, and the lipid classes mainly responsible for such separation effect were preliminarily explored. Among the various classes of lipids that differ in this comparison between treated subgroups and placebo group (Fig. 4B, lower panels), diacylglycerophosphocholines (PC) and monoacylglycerophosphocholines (LPC) displayed an interesting behavior (Fig. 4C). Concerning the PC class, an opposite behavior was observed, with levels of PC that increased upon DHA-VD treatment and decreas upon DHA-CHO-VE treatment, compatible with different effects of specific components in the multivitamin formulations. Concerning the LPC class, one of the most abundant in the human lipidome, several lipid species were present at lowered levels in the subgroups of DHA-treated patients, compared with placebo group, and especially in DHA-CHO-VE subgroup. This difference was not specifically related to PUFA-containing LPCs, since saturated and unsaturated fatty acids were equally represented in this lipid class of the different groups of patients. However, when AA was searched in all lipid species (Fig. 4C, left upper panel), significantly decreased concentrations of this PUFA were identified in the subgroups of patients on treatment with DHA-based therapy, but not in placebo-treated patients, confirmative of targeted lipidomics findings. Moreover, focusing on main LPC species, LPC (16:1) and LPC (20:4), but not LPC (22:6), significantly decreased by the effect of DHA-based therapy (Fig. 4C, right upper panel and lower panels).

## Discussion

Findings in the present study demonstrate that the improved liver histology and echogenicity of DHA-CHO-VE and DHA-VD trials^17,18^ is associated with stable and characteristic changes of patient’ plasma lipidome. Such changes include as main indicator an improved balance between the ω-6 free fatty acid AA and the administered ω-3 species DHA.

Limits of this post-hoc laboratory investigation include baseline patient’ characteristics and randomization criteria adopted to create the study groups. Essentially the bank of sample and the original randomization strategy of the clinical trials did not provide the same baseline AA/DHA levels in the different groups of patients included in this study (this ratio was higher in the DHA-based treatment group compared to the placebo group; Table 2). If on one hand that difference could represent a limit for statistics evaluations, on the other hand, it highlights the importance of investigating that specific laboratory indicator in selecting therapies as well as in diagnostics and clinical monitoring of patients; even greater seems to be the relevance of this indicator to verify the compliance to DHA therapy at the individual level. Worth of note, these limits may even reinforce the evidence of efficacy for the proposed DHA-based multivitamin treatments if we consider that, compared to placebo group, DHA-treated patients start from worst baseline levels of the proposed lipidomics indicators (such as DHA, AA, VE and some of their oxidation metabolites) to achieve an improvement that was absent in the placebo group.

Other limits are associated with the fact that lipidomics studies have been performed only in plasma, and that the number of parameters investigated with the available targeted lipidomics protocol was restricted to a small number of lipid species.

### Free PUFAs and AA/DHA ratio

The efficacy of a daily dose of 250 mg of DHA for 6 months or longer in the therapy and management of pediatric NAFLD was previously reported^14,15^ and the DHA-CHO-VE trial demonstrated that the same dose of DHA and schene of intervention can be used in combinatorial multivitamin protocols to treat pediatric NASH^18^. A dose of 500 mg was found to produce the same results in these patients when combined with 800 mg of VD in the DHA-VD study^17^. Here, we demonstrate that 6-months of therapy with DHA, independently from the dosage, stably modifies the concentrations of this and other ω-3 FA at follow-up, such as its precursor ALA. theThis results in a significant decrease of the free form of ω-6 AA, and untargeted lipidomics data confirmed lowered concentrations of AA also in complex lipids of patients on the DHA-based therapy.

However, DHA levels maintained a positive correlation with the levels of AA and the other ω-3 FFA during the study, which is expected from the converging mechanisms of biosynthesis and catabolism of these lipids^10,19^.

**Figure 5:**
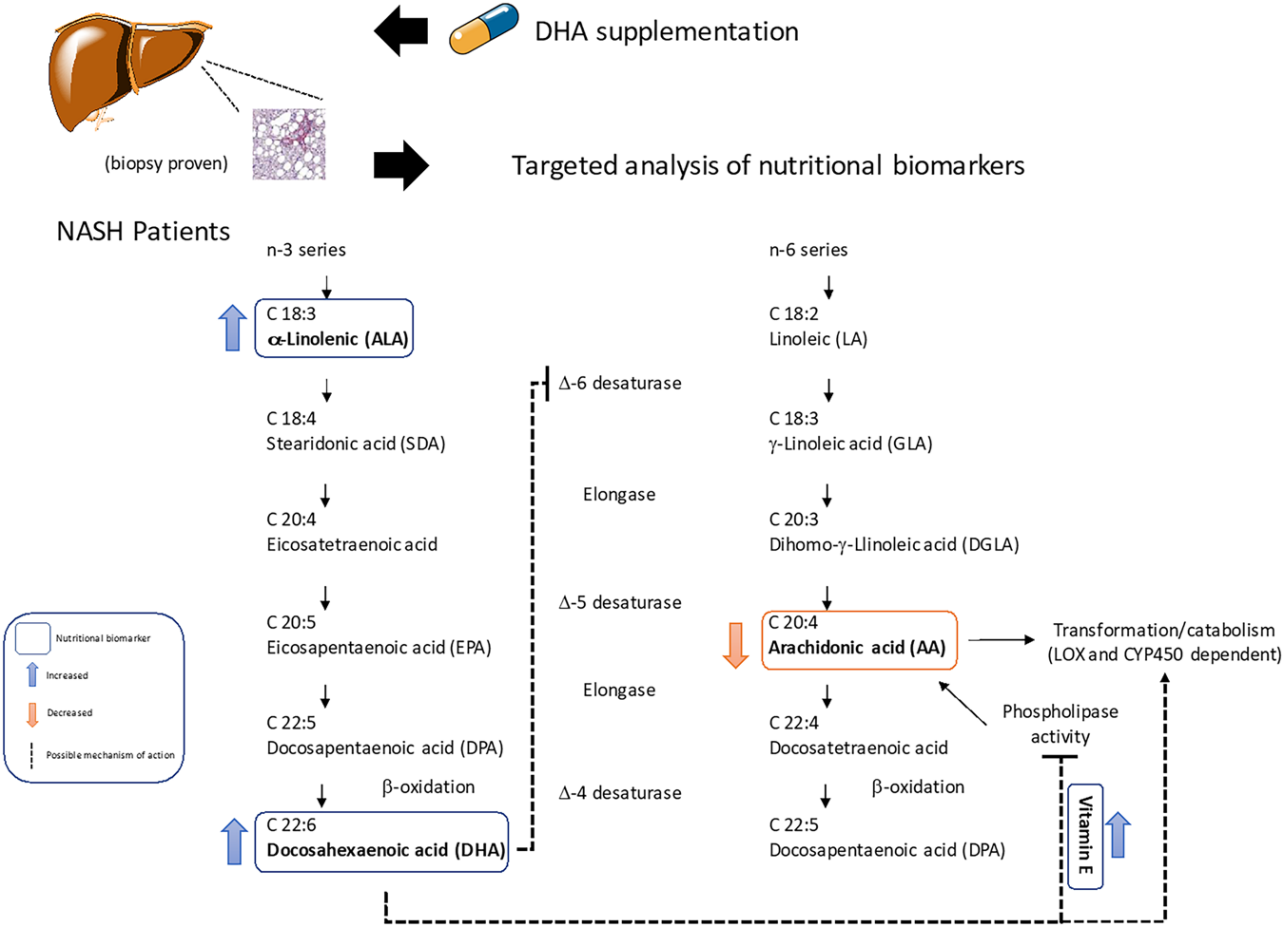
Proposed effects of the DHA-based multivitamin therapy on PUFA metabolism of pediatric NASH patients.

In this context, the AA/DHA ratio was found to represent the strongest indicator of the radiographic and histological improvements of the liver observed after the DHA-based treatment. Because the improved AA/DHA ratio was the result of non-consensual modifications in the absolute concentrations of DHA and AA (DHA increased and AA decreased post-treatment), it is conceivable speculating that the hepatic elongation and desaturation metabolism of these FA may represent a target of this therapy^20^. The competition of Δ-5 and Δ-6 desaturase activity for the precursors of DHA and AA biosynthesis is likely to be involved in the response mechanism of both the DHA-based multivitamin trials investigated in this study, and the increased concentrations of the DHA precursor ALA in the treated patients further support such hypothesis (Fig. 5). In other words, an increased availability of DHA may feedback on biosynthesis precursors of the ω-3 series ultimately lowering the entrance of ALA in this pathway. A lower demand of Δ-6 desaturase activity and a higher competition of ALA with the ω-6 biosynthesis initiator linoleic acid (18:1) for the desaturation reaction, could represent other DHA-dependent effects on this pathway that ultimately influence AA availability and its engagment in enzymatic and free radicaldependent reactions (Fig. 5). The importance of these effects is even higher if we consider that the activity of these enzymes, and especially of Δ-5 desaturase, appears to be defective in NAFLD and NASH^20,21^, leading to a poor control of DHA availability and modulatory function on insulin homeostasis and inflammatory processes of tissues^22,23^.

To our knowledge, this is the first study to identify an altered ω-6 to ω-3 fatty acid ratio in plasma and to report on its therapeutic correction in pediatric NASH. Other studies reported on an increased ω-6/ω-3 ratio of the liver as key pathogenic cue of NAFLD^24^, and recent investigation of the hepatic lipidome confirmed a pathogenic role in NASH^25^.

An unbalanced generation rate and activity of AA-derived eicosanoids and ω-3-derived anti-inflammatory metabolites, are the former and more characterized consequences of this alteration, but other mechanistic aspects, including inflammatory receptor and transcription factors, could help explaining the clinical benefit of achieving a better ω-6/ω-3 in pediatric patients, particularly if DHA levels are increased as a consequence of a nutritional intervention^10,13^.

### AA metabolism

Targeted and untargeted lipidomics data demonstrate that DHA therapy modifies the levels of AA in pediatric NASH patients, consistent with an improved control of AA metabolism. Of the AA metabolites investigated with the targeted approach, the cytochrome P450-derived ω-oxidation product 20-COOH-AA was selected as a catabolism indicator, while 20-HETE was selected to mirror the availability of this ω-6 for enzymatic transformation to bioactive eicosanoids, being this vascular mediator primarily formed through the ω-hydroxylase activity of CYP4F2 on AA^26,27^. The fact that the DHA-based multivitamin therapy significantly decreased the concentration of 20-HETE, but not that of 20-HETE/AA, also increasing average concentrations of 20-COOH-AA, is suggestive of a stimulation of AA catabolism combined with an inhibition of the enzymatic generation of inflammatory and vasoactive eicosanoids, such as 20-HETE. Although undermined by an artefactual increase due to unsuitable sample storage conditions, also the decrease LTB4 concentrations of treated patients (data not shown), point to that conclusion.

Of particular interest is the combination of DHA with the fat-soluble antioxidant vitamin E (that is further discussed below). This may influence the pathogenic interaction of AA-derived lipid mediators with other molecular players of liver lipotoxicity that derive for example from lipid peroxidation^28^ and other free radical-dependent processes of damage to liver biomolecules^11^.

Important enough, the eicosanoid metabolites selected in this targeted lipidomics study appear to be robust biomarkers useful to investigated AA catabolism/transformation in trials. 20-HETE does not seem to be as sensitive as LTB4 to pre-analytical interferences related with sample preservation and manipulation (described in detail in^29^). Indeed, its concentrations in patients with NASH and controls were much lower and close to those reported in pediatric cohorts of healthy subjects and patients affected by inflammatory diseases^30^. The same is for 20-COOH-AA that showed concentrations in the same, or even lower, range reported in adult NAFLD and NASH patients^31^.

The efficacy of DHA therapy in modifying AA metabolism lowering its distribution in complex lipids, such as LPCs, may suggest a better control of lipotoxicity and inflammatory effects of this and other classes of lipids [ref.^32^, and further discussed in^21^], an hypothesis that is worth of further investigation.

### Vitamin E status and metabolism

The increased (absolute and cholesterol-corrected) concentrations of α-TOH and those of the CYP450-derived hepatic metabolite α-CEHC, together demonstrate an improved status and consequently a higher hepatic availability and metabolism of this form of vitamin E in the treated patients^9,33^, which are stable effects maintained over the 6-month follow up phase of the study. Such conclusions are further supported by the effect of the treatment on γ-TOH and γ-CEHC concentrations that significantly decreased. This opposite effect on α-TOH and γ-TOH status can be explained by the activity of specific binding proteins in the liver such as the α-tocopherol transfer protein. These proteins show much higher affinity for α-TOH than for other forms of vitamin E, then driving the preferential cellular uptake of α-TOH and its transferring with nascent VLDL to the blood and all other extra-hepatic tissues, while the non-alpha forms (such as γ-TOH) and the quota of α-TOH exceeding the binding capacity of the liver are excreted (further details on the hepatic metabolism of vitamin E are reported in^34,35^). This effect of DHA-based therapy on α-TOH status and metabolism, is a key finding of this study because α-TOH is by far the most abundant and important chain breaker of cellular membranes and lipoprotein particles^36^; therefore, an increased availability of this vitamin is very likely to result in an increased protection of tissue PUFAs from the damaging activity of peroxyl radicals. α-TOH is also a key regulator of lipid peroxide-mediated signal transduction through inflammatory pathways, being capable of modulating the activity of cellular lipoxygenases [reviewed in^37,38^]. Again, the CYP-450 derived long-chain metabolites of α-TOH have recently been identified to possess anti-inflammatory and anti-atherogenic activity, and to represent candidate agonists of the nuclear receptors Pregnane X Receptor (PXR) and Peroxisome Proliferator-activated Receptor gamma (PPAR γ) that influence the transcription of a number of detoxification and lipid metabolism genes in the liver and other organs [reviewed in^9,35^].

The importance of improving the hepatic and lipoprotein metabolism of α-TOH in NAFLD and NASH is even more important if we consider that such diseases cause a major defect in this specific metabolism. Pioneering studies by *Nagita* and *Ando*^39^ demonstrated how the hepatic concentrations of α-TOH linearly follow those of total lipids or triglycerides accumulating in the liver of both pediatric and adult patients. These findings are in line with earliest findings obtained in animal models [ref.^39^ and references therein] and with recent data from some of us^40^. In this respect, cellular metabolism studies demonstrated how lipid accumulation promotes the retention of α-TOH in perinuclear lysosomal bodies of human hepatocytes, and such accumulation results in the stimulation of the cellular detoxification response and consequently of α-TOH ω-hydroxylase and other enzymatic players of vitamin E metabolism that sustain LCM and CEHC formation^40^.

Important enough, an improved α-TOH metabolism was confirmed in both the subgroups of pediatric NASH patients of the DHA-CHO-VE and DHA-VD trials, thus excluding a direct effect of vitamin E supplementation in such nutritional outcome.

### Combinations with choline (CHO) and vitamin D do not influence lipidomics parameters

The micronutrient formulation administered to NASH patients in one of the subgroups of patients considered in this study (namely the DHA-CHO-VE trial) also included CHO. The choice of this combination relies on the evidence that CHO intake influences the hepatic metabolism and circulating concentrations of free DHA and DHA-containing phospholipids^41^. Furthermore, defects in the important functions of this compound have been described in NAFLD/NASH patients^42^. For example, CHO administration has been reported to increase the DHA to DPA ratio within phosphatidylcholine and phosphatidylethanolamine pools of plasma^43^, a defect of which is believed to represent a lipidomic signatures of the impaired FA elongation and desaturation metabolism of the fatty liver along with increased (relative to palmitate and absolute) concentrations of the monounsaturated species palmitoleate (16:1 n7) and oleate (18:1 n9)^44^. Furthermore, the presence of CHO in multivitamin supplements containing vitamin E may help to increase the hepatic availability of this vitamin^45^.

In the present study, CHO concentrations remained unchanged during the study in DHA-CHO-VE subgroup, thus pointing to a minor influence of this micronutrient on laboratory findings. However, whether an increased intake of CHO during the 6-month intervention span of the trial sustained the positive effects of DHA and vitamin E on study endpoints, was not specifically investigated, but cannot be ruled out. In this respect, the DHA-CHO-VE multivitamin formulation produced specific effects on the phospholipidome of our patients in association with a correction of the relative abundance of 16.1 and 20:4 acyl residues esterified on LPC (Fig. 4), consistent with a positive impact on the altered lipididome of these patients^44^. Furthermore, what is sure is that a deficient intake of this vitamin-like compound should be avoided because it may lead to develop a steatogenic phenotype in animal models and humans^46,47^ and thus its co-administration with DHA and other micronutrients has to be taken under due consideration during patient management.

The correction of vitamin D deficiency in the subgroup of patients of the DHA-VD trial is considered an important goal in the clinical management of pediatric NASH^17^, but it does not appear to influence the response of PUFA and vitamin E parameters, as well as of semi-untargeted data, assessed after treatment. Again the effects of DHA therapy was similar in this subgroups of patients co-treated with VD and in the DHA-CHO-VE trial. Together these findings may lead to the same type of conclusion drawn before for CHO combination.

## Conclusions

The findings in this study demonstrate that liver echogenicity and histology improvements in pediatric NASH are produced during DHA-based therapy protocols in association with changes of ω-3 and ω-6 PUFA’ metabolism. Targeted and untargeted lipidomics indicators of this response have been identified, some of which have been measured for the first time in these patients that can be used to monitor patient’ outcome. These include plasma DHA and even more significantly its ratio with the ω-6 AA, i.e. AA/DHA. Changes in the elongation and desaturation pathways of hepatic FA may help to explain the observed results in DHA-treated patients, in association with a better control of ω-hydroxylation and catabolism of AA (Fig. 5). Vitamin E levels and metabolism are also affected by the therapy with DHA, suggesting other, and so far unexplored, therapeutic mechanisms for this ω-3 species that are under investigation in our laboratories.

In conclusion, targeted and untargeted lipidomics findings in this study demonstrate the importance of achieving a better hepatic metabolism of DHA, and consequently of its metabolic counterpart, the ω-6 AA, to treat obesity–linked pediatric NASH with the diet and micronutrient therapy.

## Methods

### Study design and laboratory investigation

The bank of samples of the DHA-CHO-VE^18^ and DHA-VD^17^ trials (registered the 08/30/2013 and 03/24/2014 at ClinicalTrials.gov with id number NCT01934777 and NCT02098317, respectively) was used to randomly selected samples in each group of this study that includ: (1) DHA-CHO-VE group (n = 15) in which patients were treated daily for 6 months with the multivitamin formulation Pro DHA Steatolip Plus® (250 mg of DHA, 39 UI of vitamin E and 201 mg of CHO); (2) DHA-VD group (n = 12) in which patients were treated daily for 6 months with the mixture of vitamin D (800 IU) and DHA (500 mg); and (3) placebo-control group (n = 15) in which patients were treated for the same time of cases with placebo. Samples of placebo group were available only from the bank of the DHA-CHO-VE trial.

During the treatment and for further 6 months of follow-up all patients were recommended to follow a hypocaloric diet (25–30 kcal/kg/day) and to engage in 1-hour physical activity twice weekly.

All children referred at the Hepato-metabolic Disease Unit of Bambino Gesù Children Hospital (Rome, Italy). The Ethics Research Committee of the Bambino Gesù Children Hospital approved the study, in accordance with the Declaration of Helsinki (as revised in Seoul, Korea, October 2008). Parents of the included patients gave their written informed consent to therapies, performed tests and publication of study results.

As described in detail in^17,18^, the primary endpoint of the two trials was the improvement in liver hyperechogenicity, expressed as ultrasound score (US), in the treatment group compared with placebo group. The US included 4 levels: normal liver echotexture (grade 0), that is without steatosis, then followed by mild (grade 1), moderate (grade 2) and severe steatosis (grade 3).

Liver biopsy was obtained to confirm the diagnosis of NASH and was repeated at the end of the study. As specified in^18^ the liver biopsy was repeated at 12 months for ethical consideration only in the DHA-CHO-VE group, whereas in the patients of placebo group associated to DHA-CO-VE trial liver histology was obtained at different times after the conclusion of the study (between 14 and 18 months) following clinical recommendations. Echo-guided liver biopsies were fixed in 10% buffered formalin and interpreted by an experienced pathologist according to criteria proposed by NAFLD Clinical Research Network^48^. Histology data were evaluated as follows: steatosis was graded 0–3 (0 = <5% steatosis, 1 = 5–33%, 2 = 33–66% and grade 3 = >66%); lobular inflammation was scored based on the number of inflammatory foci per 200x per field (0 = no inflammatory foci, 1 = <2 foci; 2 = 2–4 foci and 3 = >4 foci); ballooning was graded 0–2 (0 = none; 1 = few balloon cells present and 2 = prominent ballooning). Portal inflammation (0 = no PI, 1 = mild PI, 2 = more than mild) as previously defined^49^. Fibrosis was staged 0–4 as follows: 0 = no fibrosis; 1 = periportal or perisinusoidal; 1A = mild, Zone 3, perisinusoidal; 1B = moderate, Zone 3, perisinusoidal; 1C = portal/periportal; 2 = perisinusoidal and portal/periportal; 3 = bridging fibrosis and 4 = cirrhosis. A NAFLD activity score (NAS) >5 was used for further comparisons with variables of interest.

Secondary endpoints were correction of liver, anthropometric and metabolic parameters in both the arms of the trial. Weight was measured by a conventional scale with a precision of 100 g and height was measured by a Harpenden stadiometer with a precision of 1 mm. Body mass index (BMI) was expressed in kilograms per square meters (kg/mq). Waist circumference (WC) was evaluated by using a tape to the nearest 0.5 cm measure, at the narrowest circumference between the lower costal margin and the iliac crest in standing position.

Blood samples were collected at baseline and at month 12 after an overnight fasting and immediately processed to perform the analysis of alanine aminotransferase (ALT), aspartate aminotransferase (AST), gamma-glutamyl-transferase (GGT), total triglycerides and cholesterol, low-density lipoprotein (LDL), high-density lipoprotein (HDL), glucose and insulin by standard laboratory methods. Insulin resistance was evaluated by the homeostatic model assessment (HOMA) equation, as follows: [fasting insulin (μU/mL) X fasting glucose (mg/dL)/405]^50^. A blood tube was used to separate plasma that was divided in aliquots stored at −80 °C for further determinations that included the quantitative colorimetric determination of CHO (ABNOVA; Taiwan).

Symptoms and side effects were assessed in each visit by the investigator, who is blind to medication assignment. Complete medical histories were recorded for all participants. Collection of anthropometrical data, biochemical data and second biopsy were performed at baseline and after 12 months from trial start. Patients and investigators were blinded before and after the assignment of the intervention.

Data and samples were also obtained for a group of healthy pediatric subjects (n = 10) that were admitted at the Hepato-metabolic Disease Unit of Bambino Gesù Children Hospital (Rome, Italy) in the same period of the DHA-CHO-VE trial; exclusion criteria for these subjects were overweight/obesity and any laboratory and US sign of liver diseases.

Parents of the included patients gave their written informed consent to use data and samples for research purpose.

### Targeted lipidomics: determination of tocopherols, polyunsaturated fatty acids (PUFA) and metabolites

α-tocopherol (α-TOH), γ-tocopherol (γ-TOH) and the free form of the AA, EPA, DHA and α-linolenic acid (ALA), were measured simultaneously by LC-MS/MS using 50 µL of plasma. Metabolites of tocopherols and AA were extracted from another aliquot of plasma (300 µL) and measured in a separate run after enzymatic deconjugation. The analytes were quantified by isotopic dilution method and further details of these analytical procedures are reported as Supplementary Material.

### Untargeted lipidomics

Lipids from plasma samples were extracted in a methanol:MTBE:chloroform (MMC) mixture (40/30/30, v/v/v)^51^, containing the antioxidant BHT (10 μg/100 mL). 1000 μL of MMC solution was added to 50 μL of plasma, each sample was then vortexed and placed in a shaker at room temperature for 30 minutes (950 rpm). Samples were then centrifuged to pellet proteins (10 minutes, 8,000 rpm), and the supernatant saved for analysis in fresh Eppendorf tubes. A volume of 2 μL of each sample was injected into the LC-MS instrument.

The LC-MS system consisted of a binary pump, thermostated autosampler, and column compartment, all Dionex Ulimate 3000 series modules (Thermo Fisher Scientific, Waltham, MA USA) and a Thermo Q-exactive mass spectrometer (Thermo Fisher Scientific, Waltham, MA USA). Liquid chromatography separation was performed at 45 °C using a revers phase column, Kinetex F5 (Phenomenex inc.), at a flow rate of 0.65 ml/min. The mobile phases consisted of 5 mM ammonium formate and 0.1% formic acid in water and 5 mM ammonium formate and 0.1% formic acid in isopropanol. A gradient elution was used for the lipid separation as follows: time 0 min, solvent A 80%, B 20%; time 3 min, solvent A 60%, B 40%; time 16 min, solvent A 40%, B 60%; time 16.5 min, solvent A 30%, B 70%; time 24 min, solvent A 26%, B 74%; time 28 min, solvent A 5%, B 95%; and time 30, stop run. All solvents were purchased from Sigma-Aldrich and Biosolve (Dieuze, FR). Mass spectrometry analysis was performed first in both positive/negative ion switching method in Full MS scan mode. A preliminary untargeted data analysis with the Lipostar software (Molecular Discovery Ltd., UK)^52^ allowed to automatically generate inclusion lists for masses of interest (i.e. potential lipids). Therefore, the minimum number of samples was run again in DDS (Data Dependent Scan) positive and negative acquisition mode to obtain MS/MS spectra using the inclusion lists. Finally, MS/MS spectra were uploaded in the Lipostar session to improve lipid identification and the same software was used for multivariate statistical data analysis.

### Statistics

Study variables (presented as mean ± SD) were processed for analysis of variance by ANOVA test and differences between mean values in the experimental groups were assessed using the paired t-test of Student (two-sided test for normally distributed data) or Wilcoxon Signed rank test (for non-parametric data), when appropriate; p values < 0.05 were accepted.

Multivariable (or multiple linear) analysis was used to assess relationships between the continuous dependent variables associated with the intervention, e.g. α-tocopherol/total lipids and DHA, and other laboratory parameters assessed in the study. F values > 2.5 and p < 0.1 were considered significant.

A logistic regression model was used to assess the association between the categorical variable US or NAS and lipidomic parameters that showed significant differences in the two groups of the study; probability was assessed with the Likelihood Ratio Test (LRT). Differences were considered significant for p values ≤ 0.05 and highly significant for values ≤ 0.01.

Partial Least Squares Discriminant Analysis (PLS-DA) and Partial Least Squares Targeted Projection (PLS-TP) on semi-untargeted lipidomics data was carried out using the statistics package of the LIPOSTAR software^52^.

## Supplementary information

Online Supplemental Materials
