# Supplementary material for: Nutritional and lipidomics biomarkers of docosahexaenoic acid-based multivitamin therapy in pediatric NASH
Source: Sci Rep. 2019 Feb 14;9:2045. doi: 10.1038/s41598-018-37209-y (PMC6375912; doi:10.1038/s41598-018-37209-y)
Supplement: Supplementary file 1 — Online Supplemental Materials [file 41598_2018_37209_MOESM1_ESM.pdf]

Online Supplemental Material

Title page:

Nutritional biomarkers of docosahexaenoic acid-based multivitamin therapy in pediatric NASH.

Authors and affiliations:

Pierangelo Torquato [1, #], Danilo Giusepponi [2, #], Anna Alisi [3], Roberta Galarini [2], Desirée Bartolini [1], Marta Piroddi [1], Laura Goracci [4], Alessandra Di Veroli [4], Gabriele Cruciani [4], Annalisa Crudele [3], Valerio Nobili [5,6 §] and Francesco Galli [1 § *]

1 Department of Pharmaceutical Sciences, University of Perugia, Perugia, Italy.

2 Istituto Zooprofilattico Sperimentale dell'Umbria e delle Marche (IZSUM), Perugia, Italy.

3 Research Unit of Molecular Genetics of Complex Phenotypes, Bambino Gesù Children's Hospital, Rome, Italy.

4 Department of Chemistry, Biology and Biotechnology, University of Perugia, Perugia, Italy.

5 Hepatometabolic Unit, Bambino Gesù Children's Hospital, Rome, Italy;

6 Department of Pediatrics, Sapienza University of Rome, Italy.

# and [§] Equal contribution.

Conflict of interest statement

The author(s) declare no competing interests

*** Corresponding Author:**

Francesco Galli, PhD

University of Perugia

Department of Pharmaceutical Sciences

Lab of Clinical Biochemistry and Nutrition

Via del Giochetto, 06126 Perugia (Italy)

Tel +39 075 585 7445/7940; e-mail: francesco.galli@unipg.it

Online Supplemental Material

Materials and Methods

Reagents and standards

All HPLC-grade solvents. analytical reagents. beta glucuronidase from Escherichia Coli (G7396). sulphatase from Helix Pomatia (S9626). α-tocopherol (α-T). d_3-γ-tocopherol (d_3-γ-T) and d_6-α-tocopherol (d_6-α-T) were from Sigma Aldrich (St Louis. MO. USA). γ-tocopherol (γ-T) was purchased from Cognis Corporation (BASF. Ludwigshafen. Germany). 2. 5. 7. 8-tetramethyl-2-(2'-carboxyethyl)-6-hydroxychroman (α-carboxyethyl hydroxychromanol. α-CEHC). d_3-α-CEHC and 2. 7. 8-trimethyl-2-(2'-carboxyethyl)-6-hydroxychroman (γ-carboxyethyl hydroxychromanol. γ-CEHC) were kindly gifted by Eisai Corporation (Tokyo. Japan). α-13′-(6-hydroxy-2.5.7.8.-tetramethylchroman-2-yl)-2.6.10-trimethyltridecanol (13′-hydroxychromanol. α-13'-OH). α-13′-(6-hydroxy-2.5.7.8.-tetra-methylchroman-2-yl)-2.6.10-trimethyltridecanoic acid (13′-carboxychromanol. α-13'-COOH) and d_4-α-13'-COOH were synthetized as previously described in [1] or were kindly provided by Dr. Francesco Mazzini. Prof. Stefan Lorkowki and Prof. Marc Birringer. Deuterated and native standards of PUFAs and oxylipins were from Cayman Chemicals (Ann Arbor. MI. USA) and included: arachidonic acid (AA). arachidonic acid-d_8 (d_8-AA). α-linolenic acid (ALA). α-linolenic acid-d_5 (d_5-ALA). eicosapentaenoic acid (EPA). docosahexaenoic acid (DHA). leukotriene B4 (LTB4). leukotriene B4-d_4 (d_4-LTB4). 20-carboxy leukotriene B4 (20-COOH-LTB4). 20-hydroxy leukotriene B4 (20-OH-LTB4). 20-hydroxyeicosatetraenoic acid (20-HETE). 20-hydroxyeicosatetraenoic acid-d_6 (d_6-20-HETE) and 20-carboxy arachidonic acid (20-COOH-AA). Individual stock and working solutions of the analytical standards were prepared in methanol and stored at -80 °C.

Sample preparation

Tocopherol and PUFA metabolites – Three hundred microliters of plasma were placed in a 15 mL polypropylene tube and spiked with 50 μL of ascorbic acid (10 mg mL^{-1}). followed by 100 μL of a solution containing beta-glucuronidase (10000 U/mL) and sulfatase (200 U/mL) in 0.25 M sodium acetate. The sample was incubated at 34 °C for 30 min and. then. spiked with the internal standards d_4-α-13'-COOH. d_3-α-CEHC. d_4-LTB4 and d_6-20-HETE. After vortexing. 100 μL of glacial acetic acid and 1 mL of ethanol were added. The sample was extracted with 5 mL of a hexane/ tertiary butyl methyl ether (TBME) 2/1 v/v containing 50 mg L^{-1} butylated hydroxytoluene (BHT). After vortexing and centrifugation. the organic layer was collected into a clean glass tube. Extraction was repeated with 5 mL of a hexane/TBME mixture (1/2 v/v) containing 50 mg L^{-1} BHT. The recovered organic phases were dried under nitrogen stream. The analytes were then dissolved in 150 μL of a mixture methanol/water (75/25 v/v) containing 1 mg L^{-1} of BHT and injected for LC-MS/MS analysis. The quantification was achieved using matrix-matched calibration standards as detailed for tocopherol metabolites in [1].

Online Supplemental Material

Tocopherols and PUFAs – The sample preparation procedure of [1] was used here with some minor modifications. Briefly. 50 µL of plasma were placed in a 15 mL polypropylene tube and spiked with the isotopologues of α-T. γ-T. AA and ALA (d_6-α-T. d_3-γ-T. d_8-AA and d_5-ALA) that were used as internal standards. The extraction was carried out with 5 mL of a hexane/TBME mixture (4/1 v/v) containing 50 mg L^{-1} of BHT. After vortexing and centrifugation (3220 x g for 5 min at 10°C). the organic layer was collected into a clean glass tube. Extraction was repeated twice and the recovered organic phases were dried under a stream of nitrogen and resuspended in 500 µL of methanol containing 1 mg L^{-1} of BHT. An aliquot of the extract was further diluted to assess the analytes present at highest concentrations (double injection). Compound quantification was performed by calibration curves in methanol using the internal standardization and isotopic dilution method.

LC-MS/MS conditions

Liquid chromatography separation and mass spectrometry detection were performed on a Finnigan Surveyor LC pump system combined with a triple quadrupole mass spectrometer (TSQ Quantum Ultra. Thermo Fisher. Palo Alto. CA. USA). The separation of metabolites was achieved using a Gemini C18 column (100 mm x 2.0 mm. 3.0 µm. 100 Å. Phenomenex. Torrance. CA. USA) and water (A) and methanol (B) as mobile phases. both containing formic acid (0.1 %). For the separation of tocopherols/PUFAs. eluent A was water with 0.01% of formic acid and eluent B methanol. both containing ammonium formate (0.1 mM). The separation gradient was initiated with 50% eluent B for 1 min. followed by a linear increase up to 100% B in 8 min; this condition was maintained for 7 min. Finally. the system returned to 50 % B in 1 min and was re-equilibrated for 8 min. The column temperature was 40°C and the sample temperature was 12 °C. The flow rate was 0.3 mL min^{-1} and the injection volume 5 µL. Collision energy parameters associated with the precursor and the selected transitions are given in Supplementary Table S3 (metabolites and TQ) and Supplementary Table S4 (tocopherols/PUFAs). The electrospray ionization source (ESI) operated in positive mode except for PUFAs (ESI-).

Online Supplemental Material

Reference

1 Giusepponi, D. *et al.* Determination of tocopherols and their metabolites by liquid-chromatography coupled with tandem mass spectrometry in human plasma and serum. *Talanta* **170**, 552-561, doi:10.1016/j.talanta.2017.04.030 (2017).

Online Supplemental Material

Supplementary Table S1. Clinical and laboratory parameters in the subgroups of pediatric NASH patients and healthy controls investigated in this study.											
**Panel A DHA-CHO-VE trial (n=15)**											
	**Baseline evaluation (T0)**					**Follow-up (T1)**					
**Code sample**	**Media**	**SD**	**median**	**Min**	**Max**	**Media**	**SD**	**median**	**Min**	**Max**	**t-test vs DHA-CHO-VE T0**
Age (yrs)	13.5	2.7	13.0	10.0	17.0	14.5	2.7	14.0	11.0	18.0	
Sex	11M/4F										
Height (m)	1.5	0.1	1.5	1.3	1.7	1.5	0.1	1.5	1.3	1.7	
WC (cm)	86.6	8.6	89.0	70.0	101.0	90.0	10.5	93.5	72.0	106.5	0.18
Body weight (kg)	63.0	17.2	69.0	33.5	84.0	66.4	15.1	69.0	37.3	82.2	0.29
BMI	27.2	3.9	29.0	19.8	31.9	27.3	5.1	28.4	17.2	37.3	0.47
Laboratory parameters											
Tot. Cholesterol (mg/dL)	154.8	35.5	144.0	116.0	257.0	154.9	26.7	147.0	125.0	219.0	0.50
HDL (mg/dL)	47.4	7.2	48.0	35.0	65.0	47.6	12.2	47.0	28.0	81.0	0.47
LDL (mg/dL)	88.4	29.2	84.5	30.0	156.0	71.9	22.5	74.5	35.0	99.0	**0.05**
Triglycerides (mg/dL)	108.4	55.5	108.0	35.0	209.0	88.2	54.0	79.5	29.0	204.0	0.17
Uric Acid (mg/dL)	5.2	1.1	5.2	3.1	6.6	5.0	1.2	5.0	3.3	7.1	0.36
AST (UI/L)	33.2	11.1	33.5	20.0	63.0	30.4	17.5	25.0	17.0	85.0	0.31
ALT (UI/L)	47.3	32.0	42.5	17.0	135.0	33.1	21.1	23.5	16.0	78.0	0.09
GGT (UI/L)	17.0	8.2	15.0	6.0	36.0	21.0	17.9	15.5	6.0	79.0	0.23
Blood glucose (mg/dL)	86.9	7.7	85.5	78.0	102.0	81.4	5.5	84.0	74.0	88.0	**0.02**
Insulin (mU/L)	22.5	15.6	18.1	6.2	71.2	21.3	14.1	17.2	6.3	65.4	0.41
HOMA index	4.8	3.5	3.8	1.3	16.0	4.1	2.7	3.4	1.2	12.3	0.28

Supplementary Table S1. Clinical and laboratory parameters in the subgroups of pediatric NASH patients and healthy controls investigated in this study.											
**Panel A DHA-CHO-VE trial (n=15)**											
**Baseline evaluation (T0)**						**Follow-up (T1)**					
US and NAS											
**Code sample**	**Media**	**SD**	**median**	**Min**	**Max**	**Media**	**SD**	**median**	**Min**	**Max**	**t-test vs DHA-CHO-VE T0**
US	2.2	0.7	2.0	1.0	3.0	1.0	0.7	1.0	0.0	2.0	**0.00**
NAS	4.2	1.1	4.5	2.0	6.0	2.9	1.5	3.0	1.0	5.0	**0.01**
Vitamin E parameters											
α-TOH (nM)	14186.9	4454.2	13978.4	6765.7	22003.6	17664.5	7248.9	15477.7	9795.2	34923.3	0.08
α-TOH/Tot lipids (nmol/mg)	57.8	20.0	57.5	25.0	85.3	74.2	31.8	66.6	47.1	165.5	0.06
α-TOH/Cho (nmol/mg)	95.2	29.7	98.2	46.0	159.4	115.6	47.3	97.5	65.9	234.4	0.10
α-TOH/TG (nmol/mg)	192.5	142.2	118.5	44.8	517.6	255.0	143.3	244.6	80.6	563.3	0.14
α-13'-OH (nM)	16.6	17.3	10.1	1.7	54.6	16.9	15.6	11.9	3.9	64.9	0.48
α-13'-OH (M3 isomer; nM)	4.9	4.4	4.0	0.2	17.6	4.9	7.1	2.1	0.5	24.3	0.50
α-13'-OH + M3 (nM)	21.5	19.1	14.9	4.0	63.5	21.8	21.0	15.0	5.2	82.7	0.48
α-13'-COOH (nM)	1.6	0.6	1.4	0.5	2.5	1.5	1.6	1.0	0.6	5.7	0.49
α-CEHC (nM)	11.7	5.0	10.8	6.7	23.7	31.9	35.5	20.1	8.1	143.8	**0.02**
γ-TOH (nM)	538.9	274.9	512.8	219.2	1218.0	356.3	120.5	339.0	205.4	613.0	**0.02**
γ-TOH/Tot lipids (nmol/mg)	4.2	2.6	3.9	1.2	9.9	3.5	1.7	3.5	1.1	8.3	0.23
γ-TOH/Cho (nmol/mg)	3.6	1.9	3.3	1.1	8.8	2.3	0.7	2.3	1.2	4.1	**0.02**
γ-CEHC (nM)	146.2	68.6	130.8	63.7	264.1	122.0	62.1	106.1	41.3	234.5	0.17

Supplementary Table S1. Clinical and laboratory parameters in the subgroups of pediatric NASH patients and healthy controls investigated in this study.											
**Panel A DHA-CHO-VE trial (n=15)**											
	**Baseline evaluation (T0)**					**Follow-up (T1)**					
**Code sample**	**Media**	**SD**	**median**	**Min**	**Max**	**Media**	**SD**	**median**	**Min**	**Max**	**t-test vs DHA-CHO-VE T0**
ω-3 fatty acids (nM)											
ALA	3350.7	2222.3	3026.2	731.7	8678.2	4613.2	1158.9	4477.6	2793.3	6365.9	**0.04**
EPA	707.1	366.7	678.5	212.4	1532.3	559.3	506.2	375.2	221.2	2005.8	0.19
DHA	3838.4	1448.4	3383.3	1808.1	5778.2	6301.3	3753.3	4703.4	2969.5	15879.3	**0.02**
Arachidonic acid and metabolites (nM)											
AA	10893.9	3127.9	12038.3	6267.8	15330.8	9271.1	2596.4	8909.1	5610.7	16580.1	0.07
20-COOH AA	8.7	5.1	9.2	1.9	20.9	9.8	5.7	8.9	2.1	22.4	0.31
20-COOH-AA/AA (x1000)	0.9	0.6	0.7	0.2	2.2	1.1	0.6	0.8	0.2	2.8	**0.00**
20-HETE	21.7	6.7	21.6	8.2	31.7	13.0	4.6	12.3	6.4	23.7	**0.00**
20HETE/AA (x 1000)	2.1	0.8	2.0	0.9	4.0	1.5	0.6	1.3	0.7	2.7	**0.03**
AA/EPA	18.7	8.1	19.0	6.0	29.9	22.1	8.6	22.0	6.4	39.8	0.29
AA/DHA	3.0	0.9	2.7	1.7	5.0	1.7	0.6	1.7	0.7	2.8	**0.00**

Online Supplemental Material

Supplementary Table S1. Clinical and laboratory parameters in the subgroups of pediatric NASH patients and healthy controls investigated in this study.													
**Panel B DHA-VD trial (n=12)**													
**Baseline evaluation (T0)**							**Follow-up (T1)**						
**Code sample**	**Mean**	**SD**	**median**	**Min**	**Max**	**t-test vs DHA-CHO-VE T0**	**Mean**	**SD**	**median**	**Min**	**Max**	**t-test vs DHA-VD T0**	**t-test vs DHA-CHO-VE T1**
Age (yrs)	11.5	1.4	11.0	9.0	13.0	**0.02**	12.5	1.4	12.0	10.0	14.0		**0.02**
Sex	4M/8F												
Height (m)	1.5	0.1	1.5	1.4	1.7	0.50	1.6	0.1	1.6	1.4	1.8	0.12	0.10
WC (cm)	84.6	5.8	84.0	76.0	95.0	0.50	86.8	6.8	88.5	78.0	98.0	0.13	0.53
Body weight (kg)	65.8	13.7	62.5	47.5	84.6	0.65	67.4	15.5	64.4	46.2	94.5	0.32	0.72
BMI	27.7	3.4	29.1	23.0	32.8	0.70	27.0	3.9	28.7	20.8	32.8	0.34	0.90
Laboratory parameters													
Tot. Cholesterol (mg/dL)	162.9	22.1	159.5	124.0	194.0	0.48	156.1	23.7	159.0	103.0	200.0	0.24	0.88
HDL (mg/dL)	45.4	8.2	44.0	34.0	57.0	0.53	45.4	7.7	45.0	29.0	55.0	0.37	0.41
LDL (mg/dL)	113.1	17.0	114.5	83.0	138.0	**0.01**	117.7	21.6	119.5	88.0	151.0	0.27	**0.00**
Triglycerides (mg/dL)	112.1	46.8	110.5	45.0	224.0	0.85	135.6	65.8	128.5	48.0	270.0	0.12	**0.04**
Uric Acid (mg/dL)	5.8	1.3	6.0	4.1	7.4	0.24	5.9	1.2	6.2	3.8	9.8	0.23	0.05
AST (UI/L)	28.0	13.8	23.5	12.0	65.0	0.30	26.9	12.8	25.0	19.0	80.0	0.32	0.90
ALT (UI/L)	31.4	12.6	26.0	19.0	57.0	0.11	24.5	6.6	22.0	17.0	110.0	0.46	0.93
GGT (UI/L)	19.0	15.1	14.0	8.0	65.0	0.69	18.4	7.9	17.5	10.0	53.0	0.35	0.97
Blood glucose (mg/dL)	82.3	6.4	82.5	71.0	93.0	0.12	88.2	13.3	83.5	67.0	122.0	0.19	0.26
Insulin (mU/L)	22.4	26.8	14.5	0.4	104.4	0.99	12.7	4.3	12.0	6.4	24.8	0.14	0.08
HOMA index	4.5	5.3	2.8	0.1	20.6	0.87	2.8	1.0	2.8	1.3	5.1	0.15	0.12

Supplementary Table S1. Clinical and laboratory parameters in the subgroups of pediatric NASH patients and healthy controls investigated in this study.													
**Panel B DHA-VD trial (n=12)**													
**Baseline evaluation (T0)**							**Follow-up (T1)**						
**Code sample**	**Mean**	**SD**	**median**	**Min**	**Max**	**t-test vs DHA-CHO-VE T0**	**Mean**	**SD**	**median**	**Min**	**Max**	**t-test vs DHA-VD T0**	**t-test vs DHA-CHO-VE T1**
US and NAS													
US	3.4	0.5	3.0	3.0	4.0	**0.00**	1.9	0.9	2.0	1.0	3.0	**0.00**	**0.01**
NAS	4.8	0.7	5.0	4.0	6.0	0.08	2.1	1.2	2.5	0.0	5.0	**0.00**	0.23
Vitamin E parameters													
α-TOH (nM)	22181.2	8825.7	22929.2	7486.2	36922.8	**0.02**	26957.3	7432.6	26066.2	13256.4	39020.6	0.06	**0.00**
α-TOH/Tot lipids (nmol/mg)	84.9	31.7	97.8	28.5	135.7	**0.03**	97.1	32.2	92.4	49.3	142.7	0.17	0.07
α-TOH/Cho (nmol/mg)	136.4	45.8	148.8	52.0	209.8	**0.02**	176.0	53.4	178.0	86.1	269.1	**0.02**	**0.00**
α-TOH/TG (nmol/mg)	245.1	133.0	221.9	62.9	509.5	0.37	239.5	125.3	211.4	94.2	483.1	0.43	0.71
α-13'-OH (nM)	10.8	5.3	8.6	3.8	19.0	0.26	6.0	2.8	4.8	3.7	12.9	**0.01**	**0.03**
α-13'-OH (M3 isomer; nM)	5.4	6.9	3.7	1.6	26.9	0.83	2.0	1.1	1.8	0.3	4.9	**0.05**	0.15
α-13'-OH + M3 (nM)	16.2	10.7	13.8	6.7	45.9	0.38	8.5	3.2	7.7	5.3	15.1	**0.01**	**0.04**
α-13'-COOH (nM)	1.6	0.8	1.4	0.5	2.7	0.99	1.5	0.5	1.5	0.6	3.6	0.35	0.80
α-CEHC (nM)	20.7	20.3	11.2	6.6	66.9	0.18	37.2	11.1	37.3	17.6	71.1	**0.01**	0.44
γ-TOH (nM)	642.8	267.9	622.1	278.5	1110.6	0.35	555.5	282.7	477.7	175.3	933.6	0.27	**0.02**
γ-TOH/Tot lipids (nmol/mg)	5.2	2.5	5.9	1.6	9.0	0.57	4.2	3.2	3.3	1.2	12.4	0.16	0.04
γ-TOH/Cho (nmol/mg)	4.0	1.6	3.9	2.0	6.8	0.30	3.8	2.4	2.7	1.1	8.4	0.43	0.59
γ-CEHC (nM)	150.0	96.6	137.2	53.0	342.5	0.91	149.9	92.0	112.8	60.3	335.6	0.50	0.36

Supplementary Table S1. Clinical and laboratory parameters in the subgroups of pediatric NASH patients and healthy controls investigated in this study.													
**Panel B DHA-VD trial (n=12)**													
**Baseline evaluation (T0)**							**Follow-up (T1)**						
**Code sample**	**Mean**	**SD**	**median**	**Min**	**Max**	**t-test vs DHA-CHO-VE T0**	**Mean**	**SD**	**median**	**Min**	**Max**	**t-test vs DHA-VD T0**	**t-test vs DHA-CHO-VE T1**
ω-3 fatty acids (nM)													
ALA	3321.9	1005.2	3265.3	1863.6	5862.2	0.97	5551.8	1301.6	5481.3	3799.3	8628.2	**0.00**	0.06
EPA	721.9	352.2	657.9	301.0	1623.5	0.92	819.2	284.6	790.2	351.9	1442.4	0.32	0.17
DHA	5397.5	2671.9	4887.1	3189.4	12706.9	0.10	7489.0	3366.4	6711.4	2651.3	14265.4	0.08	0.51
Arachidonic acid and metabolites (nM)													
AA	13101.0	3231.6	12966.5	7719.4	17507.5	0.10	9264.6	2605.9	8662.2	5534.1	13983.0	**0.00**	0.76
20-COOH AA	11.4	7.7	8.5	1.4	30.5	0.33	11.3	8.0	11.4	3.1	30.8	0.45	0.46
20-COOH-AA/AA (x1000)	1.0	1.1	0.7	0.1	4.0	0.60	1.2	0.7	1.2	0.3	3.1	0.21	0.36
20-HETE	13.6	3.9	13.0	7.5	19.2	**0.00**	11.1	2.4	11.8	5.1	15.7	0.07	0.31
20HETE/AA (x 1000)	1.1	0.5	0.9	0.7	2.2	**0.00**	1.3	0.4	1.4	0.6	2.8	0.10	0.74
AA/EPA	21.2	10.2	18.8	10.3	44.6	0.52	12.5	4.9	12.6	6.3	20.6	**0.01**	**0.00**
AA/DHA	2.7	1.0	2.6	1.3	4.6	0.45	1.4	0.6	1.2	0.7	2.4	**0.00**	0.15
Vitamin D													
Vitamin D (ng/ml)	14.4	5.6	16.5	1.8	20.0	nd	22.5	6.1	21.5	14.7	34.5	**0.00**	nd

Online Supplemental Material

Supplementary Table S1. Clinical and laboratory parameters in the subgroups of pediatric NASH patients and healthy controls investigated in this study.

Panel C Placebo arm (n=15)

	**Baseline evaluation (T0)**					**Follow-up (T1)**					
**Code Sample**	**Mean**	**SD**	**median**	**Min**	**Max**	**Mean**	**SD**	**median**	**Min**	**Max**	**t-test vs Placebo T0**
Age	13.7	2.1	13.0	10.0	17.0	14.7	2.1	14.0	11.0	18.0	0.10
Sex	8M/7F										
Height (m)	1.6	0.1	1.6	1.5	1.7	1.6	0.1	1.6	1.5	1.7	0.50
WC (cm)	90.1	10.1	92.0	69.0	109.0	89.1	9.9	90.0	69.0	109.0	0.39
Body weight (kg)	66.4	15.9	68.5	35.3	85.5	66.5	16.1	68.5	35.3	85.0	0.49
BMI	28.4	5.6	28.9	20.3	43.7	28.2	5.5	28.9	20.3	43.7	0.45
Laboratory parameters											
Tot. Cholesterol (mg/dL)	157.5	31.8	151.0	125.0	257.0	146.7	16.9	142.0	125.0	177.0	0.13
HDL (mg/dL)	45.9	7.0	47.0	36.0	62.0	48.4	7.1	48.0	39.0	69.0	0.17
LDL (mg/dL)	103.5	39.5	94.0	51.0	196.0	92.9	31.2	93.0	53.0	189.0	0.21
Triglycerides (mg/dL)	88.9	47.1	80.0	35.0	192.0	81.4	35.7	69.0	44.0	170.0	0.31
Uric Acid (mg/dL)	6.9	3.5	5.6	3.6	15.0	6.9	3.5	5.6	3.6	15.0	0.50
AST (UI/L)	34.0	21.5	25.0	14.0	102.0	37.3	42.7	24.0	18.0	189.0	0.39
ALT (UI/L)	55.6	60.2	25.0	17.0	246.0	32.9	18.0	29.0	16.0	79.0	0.09
GGT (UI/L)	19.8	14.0	17.0	10.0	68.0	20.5	10.1	18.0	12.0	51.0	0.44
Blood glucose (mg/dL)	83.1	7.9	80.0	73.0	99.0	81.0	7.1	79.0	71.0	94.0	0.23
Insulin (mU/L)	23.3	16.8	17.7	7.3	60.4	21.6	13.5	15.5	11.5	59.8	0.38
HOMA index	4.8	3.6	3.6	1.6	13.6	4.3	2.8	3.5	2.2	12.8	0.34

Supplementary Table S1. Clinical and laboratory parameters in the subgroups of pediatric NASH patients and healthy controls investigated in this study.											
Panel C Placebo arm (n=15)											
	Baseline evaluation (T0)					Follow-up (T1)					
**Code Sample**	**Mean**	**SD**	**median**	**Min**	**Max**	**Mean**	**SD**	**median**	**Min**	**Max**	**t-test vs Placebo T0**
US and NAS											
US	2.0	0.8	2.0	1.0	3.0	1.5	0.6	1.0	1.0	2.5	**0.02**
NAS	4.5	1.4	5.0	2.0	7.0	4.2	1.1	4.0	2.0	6.0	0.28
Vitamin E parameters											
α-TOH (nM)	17114.0	3675.6	16901.8	11325.9	24933.5	17926.6	6595.6	16661.9	8174.7	32171.3	0.37
α-TOH/Tot lipids (nmol/mg)	66.4	27.4	77.0	0.0	99.4	80.1	34.4	80.0	34.9	152.5	0.15
α-TOH/Cho (nmol/mg)	104.3	41.8	115.1	0.0	147.9	121.7	45.8	120.8	54.1	226.6	0.18
α-TOH/TG (nmol/mg)	198.7	95.5	213.8	0.0	302.5	255.8	140.2	251.2	69.4	480.1	0.14
α-13'-OH (nM)	11.8	10.3	9.3	1.7	42.8	10.9	6.6	10.0	1.7	24.6	0.40
α-13'-OH (M3 isomer; nM)	3.7	3.8	2.5	0.2	13.8	5.8	5.9	2.9	0.4	19.3	0.17
α-13'-OH + M3 (nM)	15.6	13.6	11.5	2.0	56.6	16.7	10.4	13.9	3.8	37.7	0.41
α-13'-COOH (nM)	1.6	0.9	1.8	0.2	2.8	2.6	1.3	2.3	1.1	5.0	**0.03**
α-CEHC (nM)	14.4	16.0	10.9	2.6	59.6	14.0	8.5	10.5	5.5	35.2	0.47
γ-TOH (nM)	485.5	184.9	533.0	144.6	743.3	447.5	133.6	438.5	239.1	717.8	0.28
γ-TOH/Tot lipids (nmol/mg)	4.4	2.4	4.9	0.4	7.4	4.6	2.9	4.9	1.3	12.0	0.40
γ-TOH/Cho (nmol/mg)	3.2	1.3	3.4	0.6	5.2	3.0	0.9	3.0	1.4	4.7	0.38
γ-CEHC (nM)	142.2	118.1	92.1	34.6	378.2	141.6	94.3	112.6	54.5	333.6	0.49

Supplementary Table S1. Clinical and laboratory parameters in the subgroups of pediatric NASH patients and healthy controls investigated in this study.											
Panel C Placebo arm (n=15)											
	Baseline evaluation (T0)					Follow-up (T1)					
**Code Sample**	**Mean**	**SD**	**median**	**Min**	**Max**	**Mean**	**SD**	**median**	**Min**	**Max**	**t-test vs Placebo T0**
ω-3 fatty acids (nM)											
ALA	4130.3	1822.9	3426.0	2563.0	8080.5	4380.3	1764.6	3975.2	2404.0	7785.3	0.37
EPA	659.5	391.2	529.9	169.7	1343.7	519.2	200.2	510.0	286.8	824.9	0.14
DHA	5537.0	1890.5	5490.0	1743.9	8920.3	4885.4	1556.5	4358.3	3089.1	7728.4	0.18
Arachidonic acid and metabolites (nM)											
AA	9911.9	3523.5	9593.8	2997.4	15959.3	9632.5	3214.6	9848.6	5543.5	16451.5	0.42
20-COOH AA	13.9	4.9	13.3	7.1	21.3	10.8	6.4	8.6	3.6	24.8	0.10
20-COOH-AA/AA (x1000)	1.6	1.2	1.3	0.7	5.1	1.2	0.7	1.0	0.3	2.6	0.12
20-HETE	18.0	8.2	17.3	6.6	31.8	13.7	6.1	12.5	6.6	24.7	0.08
20HETE/AA(x 1000)	2.0	1.1	2.0	0.7	4.1	1.5	0.6	1.3	0.6	3.0	0.07
AA/EPA	17.9	6.6	17.9	9.5	28.4	19.7	5.3	20.4	9.9	29.6	0.23
AA/DHA	1.8	0.3	1.8	1.3	2.3	2.0	0.3	2.0	1.6	2.7	0.07
Vitamin D											
Vitamin D (ng/ml)	13.9	2.9	13.9	9.1	18.2	14.2	2.6	13.9	11.0	18.1	0.42

Online Supplemental Material

Supplementary Table S1. Clinical and laboratory parameters in the subgroups of pediatric NASH patients and healthy controls investigated in this study.							
**Panel D Healthy control subjects (n=10)**							
**Code Sample**	**Mean**	**SD**	**median**	**Min**	**Max**	**t-test vs T0 (all data)**	**t-test vs treatment (DHA-based multivitamin) arm T1**
Age	12.3	3.3	12.5	7.0	17.0	0.25	0.12
Sex	6M/4F						
Height (m)	1.4	0.2	1.4	1.2	1.8	**0.01**	**0.03**
Body weight (kg)	40.2	16.9	36.7	18.5	71.0	**0.00**	**0.00**
BMI	18.3	3.4	18.4	13.2	22.9	**0.00**	**0.00**
Laboratory parameters							
Tot. Cholesterol (mg/dL)	147.3	18.0	148.5	122.0	172.0	0.14	0.17
HDL (mg/dL)	50.8	10.2	52.5	29.0	64.0	**0.05**	0.12
LDL (mg/dL)	121.8	26.3	129.5	70.0	154.0	**0.03**	**0.01**
Triglycerides (mg/dL)	81.6	41.8	71.0	41.0	192.0	0.12	0.09
AST (UI/L)	29.1	6.4	27.5	19.0	42.0	0.29	0.38
ALT (UI/L)	20.3	5.7	19.0	14.0	35.0	**0.03**	**0.05**
GGT (UI/L)	13.9	7.7	13.5	5.0	33.0	0.13	0.08
Blood glucose (mg/dL)	79.7	5.3	79.5	70.0	88.0	**0.04**	0.13

Supplementary Table S1. Clinical and laboratory parameters in the subgroups of pediatric NASH patients and healthy controls investigated in this study.							
**Panel D Healthy control subjects (n=10)**							
**Code Sample**	**Mean**	**SD**	**median**	**Min**	**Max**	**t-test vs T0 (all data)**	**t-test vs treatment (DHA-based multivitamin) arm T1**
Vitamin E parameters							
α-TOH (nM)	17755.4	3880.0	17945.4	13107.9	23055.5	0.50	0.09
α-TOH/Tot lipids (nmol/mg)	63.3	39.7	70.6	0.0	123.0	0.30	**0.05**
α-TOH/Cho (nmol/mg)	95.4	54.9	102.1	0.0	162.2	0.16	**0.01**
α-TOH/TG (nmol/mg)	209.0	160.5	217.4	0.0	510.2	0.48	0.24
α-13'-OH (nM)	17.0	13.2	12.1	3.1	43.1	0.21	0.16
α-13'-OH (M3 isomer; nM)	7.5	5.9	6.6	1.6	20.8	0.07	**0.03**
α-13'-OH + M3 (nM)	24.5	13.1	24.1	5.4	45.5	0.11	0.07
α-13'-COOH (nM)	2.3	1.8	2.3	0.1	4.5	0.06	0.13
α-CEHC (nM)	15.6	8.1	13.9	6.1	30.7	0.47	**0.02**
γ-TOH (nM)	490.3	204.4	466.9	160.3	869.4	0.24	0.34
γ-TOH/Tot lipids (nmol/mg)	3.4	1.5	3.1	1.3	6.1	0.35	0.30
γ-TOH/Cho (nmol/mg)	5.1	2.4	4.9	1.9	9.9	0.26	0.07
γ-CEHC (nM)	161.9	69.9	152.9	83.4	327.8	0.31	0.17

Supplementary Table S1. Clinical and laboratory parameters in the subgroups of pediatric NASH patients and healthy controls investigated in this study.							
**Panel D Healthy control subjects (n=10)**							
**Code Sample**	**Mean**	**SD**	**median**	**Min**	**Max**	**t-test vs T0 (all data)**	**t-test vs treatment (DHA-based multivitamin) arm T1**
ω-3 fatty acids (nM)							
ALA	4810.8	2306.6	3812.7	2156.5	8688.4	**0.04**	0.35
EPA	720.2	452.2	616.7	294.4	1783.0	0.43	0.36
DHA	4592.5	1696.3	3910.6	2603.9	7318.1	0.36	**0.04**
Arachidonic acid and metabolites (nM)							
AA	10966.6	3604.3	9910.0	5924.2	17305.5	0.42	**0.05**
20-COOH AA	9.5	5.6	8.0	2.8	18.0	0.22	0.31
20-COOH-AA/AA (x1000)	0.9	0.7	0.8	0.0	2.3	0.23	0.14
20-HETE	16.3	5.5	19.0	6.6	20.5	0.23	**0.01**
20HETE/AA (x 1000)	1.6	0.8	1.4	0.6	3.3	0.28	0.26
AA/EPA	18.7	7.5	21.3	6.3	26.6	0.44	0.37
AA/DHA	2.6	0.8	2.7	1.2	3.8	0.48	**0.00**

Online Supplemental Material

**Supplementary Table S2. Multivariable Linear Fitting of nutritional and lipidomics biomarkers investigated in this study.** Data were from the treatment arm at 12-month evaluation time point. Statistics was calculated by ANOVA test.					
**Panel A<br>Independent variable: AA/DHA**	**Statistics**			**ANOVA**	
	**Intercept**	**Slope**	**Adj. R2**	**F value**	**Prob>F**
General characteristics					
Age (yrs)	12.93241	0.35981	-0.03358	0.18778	0.66864
Height (m)	1.48605	0.03408	-0.01228	0.69681	0.41209
WC (cm)	85.72264	2.04588	-0.02262	0.44702	0.51013
Weight (kg)	61.08407	4.01354	-0.01489	0.63331	0.43394
BMI	26.30805	0.59187	-0.03505	0.15332	0.69884
Laboratory parameters					
Tot. Cholesterol (mg/dL)	171.00858	-9.75803	0.01756	1.44696	0.24075
HDL (mg/dL)	40.33085	3.64254	0.00267	1.06697	0.31193
LDL (mg/dL)	108.58901	-9.78464	-0.00601	0.85076	0.36552
Triglycerides (mg/dL)	147.53444	-22.72099	0.00652	1.16416	0.29133
Uric Acid (mg/dL)	6.13225	-0.34472	-0.02164	0.47058	0.49929
AST (UI/L)	32.5537	-1.08245	-0.04034	0.03055	0.86271
ALT (UI/L)	41.09435	-5.18478	-0.02526	0.40868	0.52896
GGT (UI/L)	23.48374	-1.50134	-0.03811	0.08223	0.77676
Blood glucose (mg/dL)	86.24758	-1.61984	-0.03276	0.20692	0.65328
Insulin (mU/L)	23.87934	-3.87234	6.12698E-4	1.01533	0.32368
HOMA index	1.52391	1.29186	0.09368	3.58405	0.07046
Vitamin E parameters					
α-TOH (µM)	32090.46021	-6241.50443	0.14612	5.27813	**0.03062**
α-TOH/Tot lipids (nmol/mg)	107.40038	-14.22621	0.02764	1.71061	0.2033
α-TOH/Cho (nmol/mg)	197.94276	-33.57724	0.08084	3.19869	**0.08633**
α-TOH/TG (nmol/mg)	280.91383	-22.0663	-0.03112	0.24548	0.62478
α-13'-OH (nM)	12.69775	-0.35814	-0.04137	0.0069	0.9345
α-13'-OH (M3 isomer; nM)	0.26406	2.09635	0.01458	1.36994	0.25331
α-13'-OH + M3 (nM)	12.96181	1.73821	-0.03766	0.09266	0.76344
α-13'-COOH (nM)	2.07147	-0.3086	-0.03225	0.37513	0.54748
α-CEHC (nM)	62.24443	-16.84827	0.09602	3.65542	**0.0679**
γ-TOH (µM)	399.87362	35.06853	-0.03298	0.20175	0.65734
γ-TOH/Tot lipids (nmol/mg)	2.34714	0.42488	-0.02015	0.50624	0.48363
γ-TOH/Cho (nmol/mg)	2.21495	0.99115	0.02174	1.55545	0.22437
γ-CEHC (nM)	133.23492	1.12033	-0.04158	0.00192	0.96542

**Supplementary Table S2. Multivariable Linear Fitting of nutritional and lipidomics biomarkers investigated in this study.** Data were from the treatment arm at 12-month evaluation time point. Statistics was calculated by ANOVA test.					
**Panel A**<br>**Independent variable: AA/DHA**	**Statistics**		**ANOVA**		
	**Intercept**	**Slope**	**Adj. R2**	**F value**	**Prob>F**
Fatty acids and metabolites					
ALA(nM)	7093.44134	-1295.43578	0.34561	14.20349	**9.43156E-4**
EPA(nM)	1324.35084	-418.9632	0.31044	12.25476	**0.00184**
DHA(nM)	13583.41088	-4345.88238	0.52141	28.23669	**1.88131E-5**
AA(nM)	9415.60422	-184.47155	-0.0398	0.04312	0.83724
20-COOH-AA(nM)	7.62537	1.95444	-0.00996	0.7535	0.39397
20-COOH-AA/AA (x 1000)	0.89392	0.20992	-0.01498	0.63102	0.43477
20-HETE(nM)	12.42566	-0.08552	-0.04148	0.00424	0.94861
20-HETE/AA (x 1000)	1.48184	-0.02632	-0.04094	0.01671	0.89822
AA/EPA	2.89111	9.39304	0.41668	18.85819	**2.21025E-4**
AA/DHA	0	1	1	--	--
Vitamin D					
Vitamin D (ng/ml)	5.39511	3.63539	-0.09985	0.00135	0.97138

Online Supplemental Material

Supplemental Table S3 – Retention times (RT) and MS/MS transitions of tocopherols and PUFA metabolites					
Analyte	RT (min)	Adduct ion	Precursor ion *(m/z)*	Product ion *(m/z)*	Collision Energy (eV)
γ-CEHC	4.6	$[M+H]^+$	265	151	18
LTB4-COOH	4.8	$[M+Na]^+$	389	353	16
				371	14
LTB4-OH	5.2	$[M+Na]^+$	375	339	14
				357	16
d_3-α-CEHC	5.2	$[M+H]^+$	282	168	16
α-CEHC	5.3	$[M+H]^+$	279	165	17
d_4-LTB4	8.0	$[M+Na]^+$	363	345	13
LTB4	8.1	$[M+Na]^+$	359	323	13
				341	12
20-COOH ARA	8.9	$[M+H]^+$	335	299	12
				317	5
d_6-20-HETE	9.0	$[M+H]^+$	327	309	9
20-HETE	9.1	$[M+H]^+$	321	285	10
				303	5
d_4-13'-α-COOH	11.4	$[M+H]^+$	465	169	28
13'-α-COOH	11.5	$[M+H]^+$	461	165	26
13'-α-OH	11.7	$[M+H]^+$	447	165	21

Online Supplemental Material

Supplemental Table S4 – Retention times (RT) and MS/MS transitions of tocopherols and PUFAs.					
Analyte	RT (min)	Adduct ion	Precursor ion (m/z)	Product ions (m/z)	Collision energy
EPA	10.8	$[M-H]^-$	301	257	15
				203	16
d_5-α-ALA	10.8	$[M-H]^-$	282	282	5
ALA	10.9	$[M-H]^-$	277	277	5
				233	17
d_8-AA	11.1	$[M-H]^-$	311	267	18
AA	11.2	$[M-H]^-$	303	259	17
				205	17
DHA	11.2	$[M-H]^-$	327	229	17
				283	15
d_3-γ-T	14.0	$[M^{\cdot}]^+$	419	154	
γ-T	14.1	$[M^{\cdot}]^+$	416	151	34
d_6-α-T	14.4	$[M+H]^+$	437	171	
α-T	14.5	$[M+H]^+$	431	165	19

Online Supplemental Material

Legend to Supplemental Figures

Supplementary Figure S1. Levels of the radiographic and histological endpoints NAS and US in pediatric NASH patients treated with DHA-based multivitamin therapy or placebo. The levels of these categorical variables in the DHA-based treatment group (DHA-VD and DHA-CHO-VE subgroups merged) and placebo group are shown in panel A. Logistic regression analysis (B and C) was used to assess the correlation between these radiographic and histological variables in the two groups of patients.

Supplementary Figure S2. Correlations between the plasma ω-3 fatty acids DHA and ALA. and the ω-6 AA. in pediatric NASH patients treated with DHA-based multivitamin therapy or placebo. A linear regression model was used to assess the relationship between the free forms of these PUFAs in the DHA-based treatment group (DHA-VD and DHA-CHO-VE subgroups merged) and placebo group.

Supplementary Figure S3. Correlations between plasma DHA and AA levels. and the radiographic and histological endpoints NAS and US in paediatric NASH patients treated with DHA-based multivitamin therapy or placebo. Logistic regression analysis of the relationship existing between NAS (A) or US (B) with the ω-3 fatty acid DHA (upper panels) and the ω-6 AA (lower panels) in DHA treated or placebo treated patients (left and right charts. respectively).

Online Supplemental Material

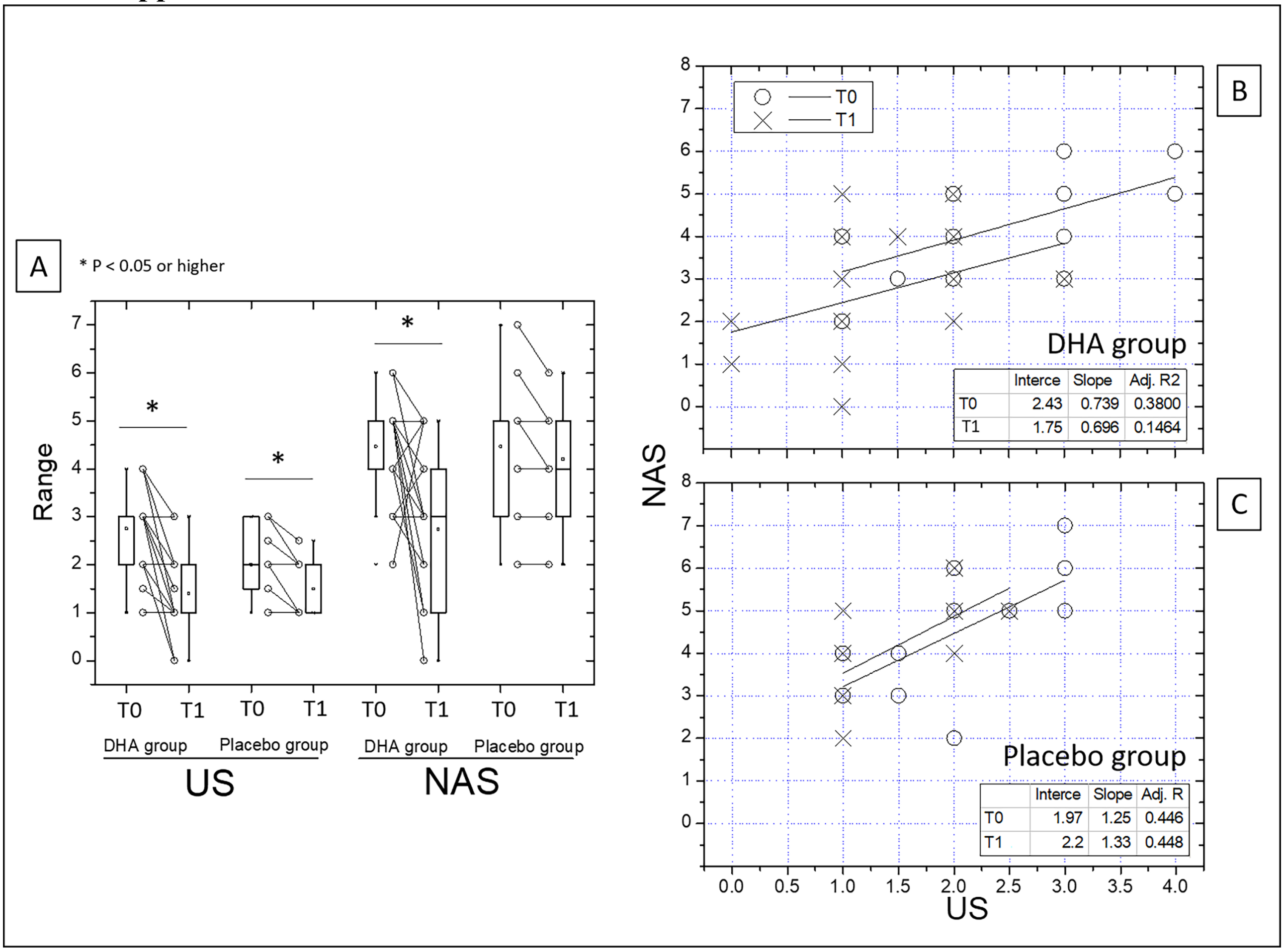

Supplementary Figure S1

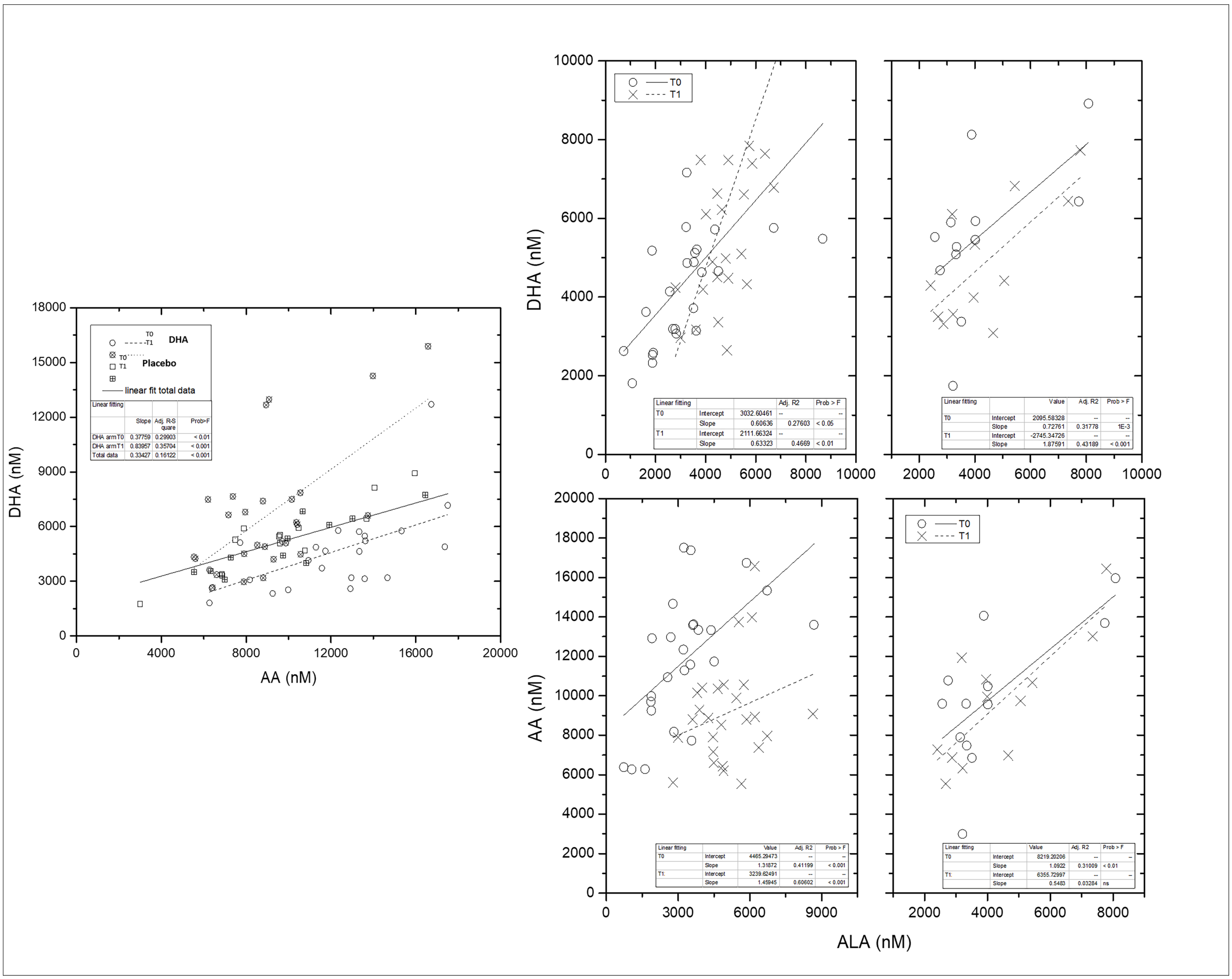

Supplementary Figure S2

Online Supplemental Material

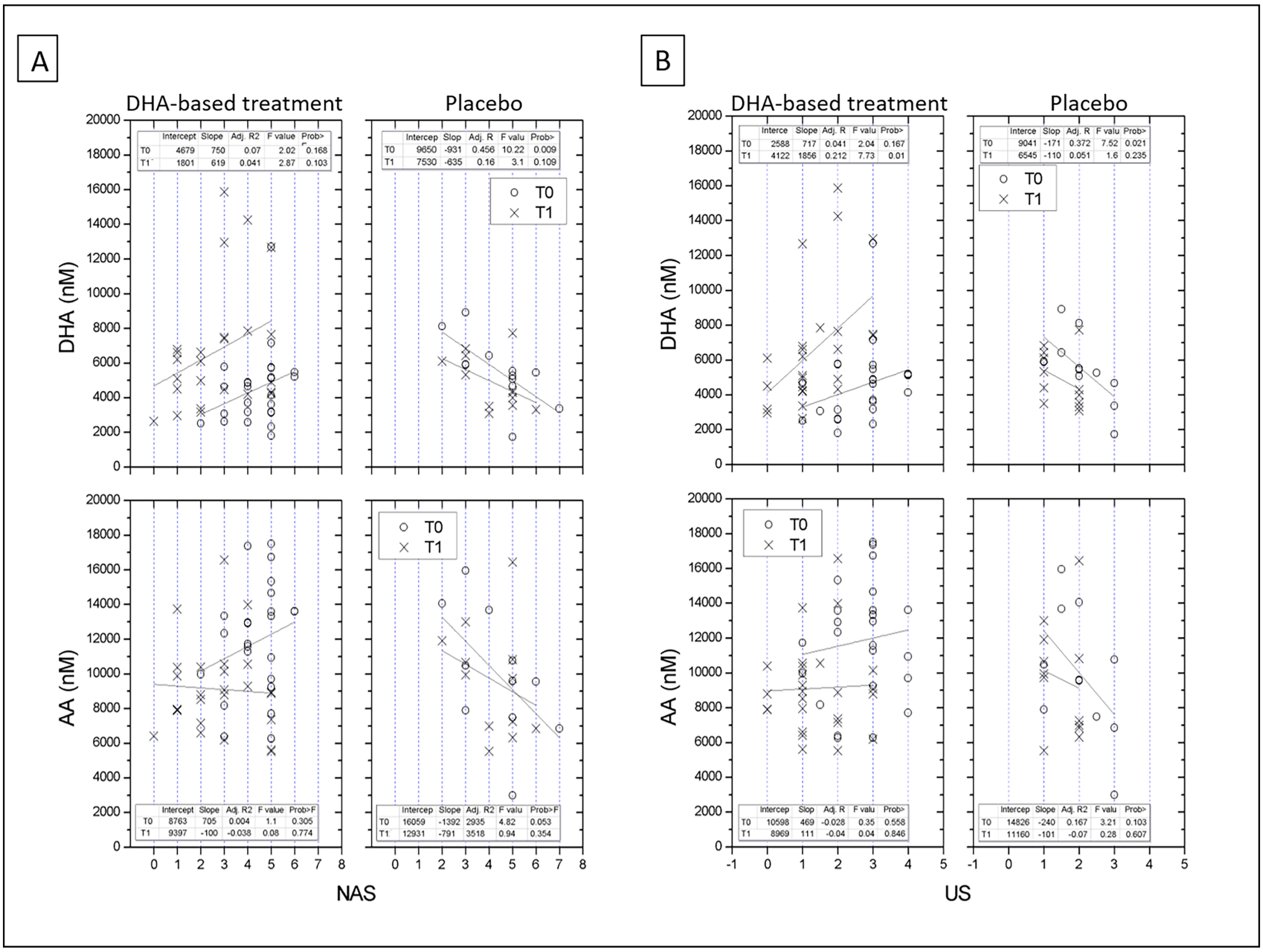

Supplementary Figure S3
